# Gene expression and DNA methylation changes in response to hypoxia in toxicant-adapted Atlantic killifish (*Fundulus heteroclitus*)

**DOI:** 10.1242/bio.061801

**Published:** 2025-01-06

**Authors:** Neelakanteswar Aluru, Yaamini R. Venkataraman, Christopher S. Murray, Veronica DePascuale

**Affiliations:** ^1^ Biology Department, Woods Hole Oceanographic Institution, Woods Hole, MA 02543, USA; ^2^ Woods Hole Center for Oceans and Human Health, Woods Hole Oceanographic Institution, Woods Hole, MA 02543, USA; ^3^ College of Arts and Sciences, Oberlin College and Conservatory, Oberlin, OH 44074, USA

**Keywords:** Mummichog, DNA methylation, Hypoxia, RNAseq, Evolved resistance, Cost of tolerance

## Abstract

Coastal fish populations are threatened by multiple anthropogenic impacts, including the accumulation of industrial contaminants and the increasing frequency of hypoxia. Some populations of the Atlantic killifish (*Fundulus heteroclitus*), like those in New Bedford Harbor (NBH), Massachusetts, USA, have evolved a resistance to dioxin-like polychlorinated biphenyls (PCBs) that may influence their ability to cope with secondary stressors. To address this question, we compared hepatic gene expression and DNA methylation patterns in response to mild or severe hypoxia in killifish from NBH and Scorton Creek (SC), a reference population from a relatively pristine environment. We hypothesized that NBH fish would show altered responses to hypoxia due to trade-offs linked to toxicant resistance. Our results revealed substantial differences between populations. SC fish demonstrated dose-dependent changes in gene expression in response to hypoxia, while NBH fish exhibited a muted transcriptional response to severe hypoxia. Interestingly, NBH fish showed significant DNA methylation changes in response to hypoxia, while SC fish did not exhibit notable epigenetic alterations. These findings suggest that toxicant-adapted killifish may face trade-offs in their molecular response to environmental stress, potentially impacting their ability to survive severe hypoxia in coastal habitats. Further research is needed to elucidate the functional implications of these epigenetic modifications and their role in adaptive stress responses.

## Teaser

**Summary:** This study reveals how evolved resistance to toxicants in killifish may compromise their ability to respond to hypoxia, highlighting trade-offs that impact survival in stressed coastal environments.

## INTRODUCTION

Adaptation to environmental stressors is critical for survival in rapidly changing ecosystems. Understanding the physiological and molecular responses that underlie adaptive mechanisms is essential for predicting organismal sensitivity. Fish populations, particularly those inhabiting coastal waters, often face multiple environmental challenges simultaneously, which can compound the stress on these organisms. One such common stressor is hypoxia – low oxygen levels in the environment – which is increasingly documented in coastal regions due to anthropogenic activities leading to excess nutrient loading, harmful algal blooms and climate change (Wallace and Gobler, 2021). The presence and accumulation of anthropogenic chemicals in coastal ecosystems poses an additional, co-occurring threat to the health of fish populations. The ability of fish to cope with hypoxia is mediated through a range of physiological, transcriptional, and epigenetic mechanisms. However, very little is known about how populations chronically exposed to toxicants respond to secondary stressors such as hypoxia.

The Atlantic killifish (*Fundulus heteroclitus*) is one of the most abundant estuarine fish distributed along the East coast of the USA, where they play an ecologically important role in the food webs (Able et al., 2007; Kimball and Able, 2007). Their ability to tolerate wide changes in environmental conditions, including temperature, salinity, oxygen and pH, have made them an ideal model species to investigate the biochemical, physiological and evolutionary basis of environmental adaptation (Borowiec et al., 2020, 2018; Borowiec and Scott, 2020). Some populations of killifish are also valuable models for understanding the mechanisms of evolved resistance to toxicants (Harishchandra et al., 2024; Nacci et al., 2010; Reid et al., 2016). Populations of killifish inhabiting contaminated coastal waters along the North Atlantic US coast have evolved resistance to some contaminants representing major categories of aryl hydrocarbon pollutants, such as polynuclear aromatic hydrocarbons (PAHs), and halogenated aromatic hydrocarbons such as polychlorinated biphenyls (PCBs), 2,3,7,8-tetrachlorodibenzo-p-dioxin (TCDD, ‘dioxin’) and other dioxin-like compounds (DLCs) (Nacci et al., 2010). This evolved resistance involves alterations in signaling through the aryl hydrocarbon receptor (AHR), a ligand-activated transcription factor that forms a heterodimer with aryl hydrocarbon receptor translocator (ARNT), binding to dioxin-response elements to regulate the expression of target genes. While there is a great deal of understanding about the physiological and biochemical basis of adaptation to a variety of environmental conditions including toxicants in this species, very little is known about the impact of resistance to toxicants on their ability to respond to subsequent stressors such as hypoxia (Jayasundara et al., 2017; Jasperse et al., 2023; Lindberg et al., 2017).

The hypoxia-inducible factor (HIF) signaling pathway is a critical cellular response mechanism that enables organisms to respond to hypoxic conditions. Under normal oxygen levels (normoxia), HIF-α subunits (mainly HIF-1α and HIF-2α) are hydroxylated by prolyl hydroxylase enzymes, marking them for degradation via the von Hippel-Lindau (VHL) ubiquitin-proteasome pathway (Fong and Takeda, 2008; Kallio et al., 1999). This prevents the accumulation of HIF-α under normoxia. Under hypoxic conditions, the activity of prolyl hydroxylases is inhibited due to a lack of oxygen, leading to the stabilization of HIF-α (Ivan and Kaelin, 2017). Once stabilized, HIF-α translocates into the nucleus, where it dimerizes with ARNT, also known as HIF-1β. This HIF-α/ARNT complex binds to hypoxia response elements (HREs) and regulates the transcription of target genes (Kindrick and Mole, 2020; Li et al., 2023). Some of the target genes regulate processes such as angiogenesis (e.g. VEGF), erythropoiesis, glucose metabolism (e.g. GLUT1), and anaerobic metabolism (e.g. LDHA) (Luo et al., 2022). Similar responses were observed in killifish, suggesting conserved physiological and molecular mechanisms across species (Borowiec et al., 2020, 2018, 2015; Borowiec and Scott, 2020, 2021; Du et al., 2016; Lau et al., 2021; Ridgway and Scott, 2023).

The AHR and HIF pathways exhibit crosstalk primarily through their shared use of the heterodimerizing partner ARNT (Vorrink and Domann, 2014). Since ARNT is a limiting factor, competition between AHR and HIF for ARNT can influence the balance of responses to environmental toxins and hypoxic stress. This crosstalk may result in altered cellular outcomes, particularly in situations where both pathways are activated simultaneously, such as under environmental stress. One study tested this hypothesis in Atlantic killifish by exposing them to a dioxin-like PCB for 3 days, followed by a hypoxia challenge (Kraemer and Schulte, 2004). Prior PCB exposure disrupted the classical hypoxia response by increasing hepatic glycolytic enzyme activity, suggesting that dioxin-induced AHR activation could limit ARNT availability for the hypoxia response.

The objective of this study was to investigate the impact of evolved resistance to toxicants on response to acute hypoxia. We characterized the hepatic gene expression and DNA methylation patterns in response to two levels of hypoxia in two distinct populations of Atlantic killifish (*Fundulus heteroclitus*). One population is from Scorton Creek, Sandwich, MA, USA (SC), a relatively pristine environment, and is considered sensitive to environmental toxicants (Bello et al., 2001). The other population originates from New Bedford Harbor, MA, USA (NBH), a superfund site heavily contaminated with dioxin-like PCBs, where the killifish have evolved resistance to toxicants. The NBH population represents a unique case study in how toxicant-adapted organisms might exhibit trade-offs in their ability to cope with additional stressors, such as hypoxia. We hypothesized that fish from NBH would exhibit altered responses to acute hypoxia compared to the sensitive Scorton Creek (SC) population. Specifically, we predicted that the NBH fish would show differential hepatic gene expression and DNA methylation patterns in response to hypoxia, potentially compromising their ability to mount an optimal response to low oxygen conditions compared to the SC population.

## RESULTS

### NBH fish are resistant to dioxin-like PCB exposure

**Fig. 1.**
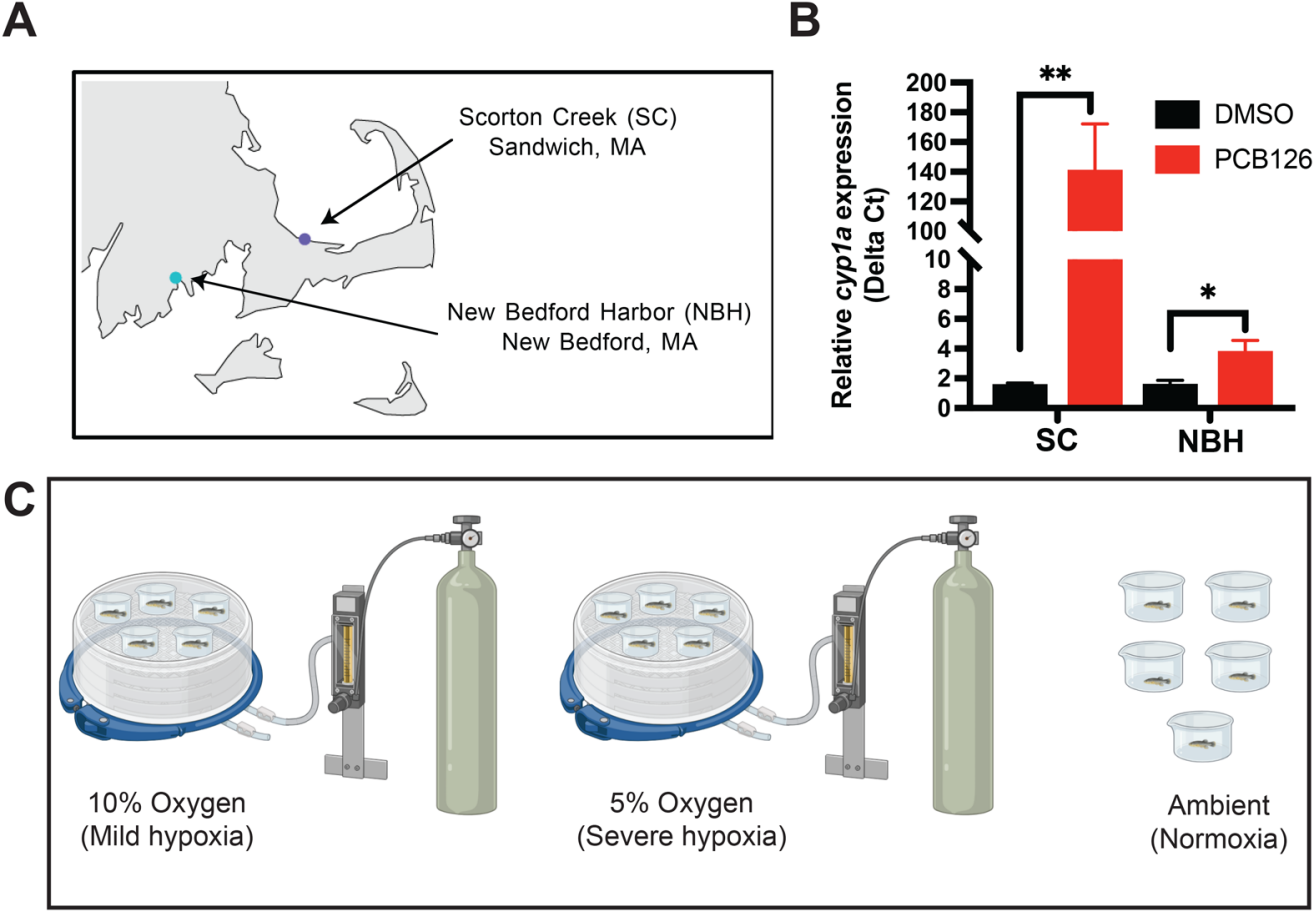
**Experimental overview.** (A) Map of Southeastern Massachusetts showing the collection sites of sensitive (SC) and resistant (NBH) Atlantic killifish. (B) Relative expression of *cyp1a* in SC and NBH fish embryos in response to PCB126 (50 nM) exposure. *** represents statistically significant difference from the control group. ** p.value = 0.0001 and * p.value = 0.01. (C) Illustration of the experimental setup. Mild and severe hypoxia exposures were conducted by pumping oxygen containing either 5% or 10% air saturation into the chambers, respectively. Control group was maintained outside under ambient conditions.

To ensure that NBH and SC fish exhibit toxicant resistant and sensitive phenotypes, we exposed the embryos from both populations to PCB126 and quantified *cyp1a* gene expression using quantitative real-time PCR. SC embryos exposed to PCB126 induced *cyp1a* gene expression several hundred-fold compared to the DMSO group suggesting sensitivity to dioxin-like PCBs. In contrast, NBH fish showed a muted response to PCB126 exposure suggesting resistance to dioxin-like PCBs (Fig. 1B).

### Hypoxia exposure did not cause loss of equilibrium

Neither mild nor severe hypoxia exposure had any effect on the loss of equilibrium during the 6-h exposure period in fish from both populations. Upon initial transfer into the hypoxia chamber, fish from both populations exhibited a rapid swimming response for the first 10-15 min. This was followed by a noticeable reduction in swimming activity accompanied by rapid ventilation (opercular movements). By the end of the exposure period, the fish were consistently found at the bottom of the container, exhibiting slow opercular movements but there was no loss of equilibrium as evidenced by their ability to maintain position and coordinated movement.

### Differential responses to hypoxia in toxicant sensitive and resistant populations

Strand-specific RNA sequencing of NBH and SC samples yielded an average of 17.2 million reads per sample. Of these, 83% of the reads were uniquely mapped to the genome. The summary of mapping statistics and read counts for annotated genes is provided in the supplementary information (RNAseq_supplementaryInformation.xlsx). Principal component coordinate analysis revealed one NBH hypoxia sample to be an outlier, which was omitted from statistical analysis (Fig. S2).

#### SC

**Fig. 2.**
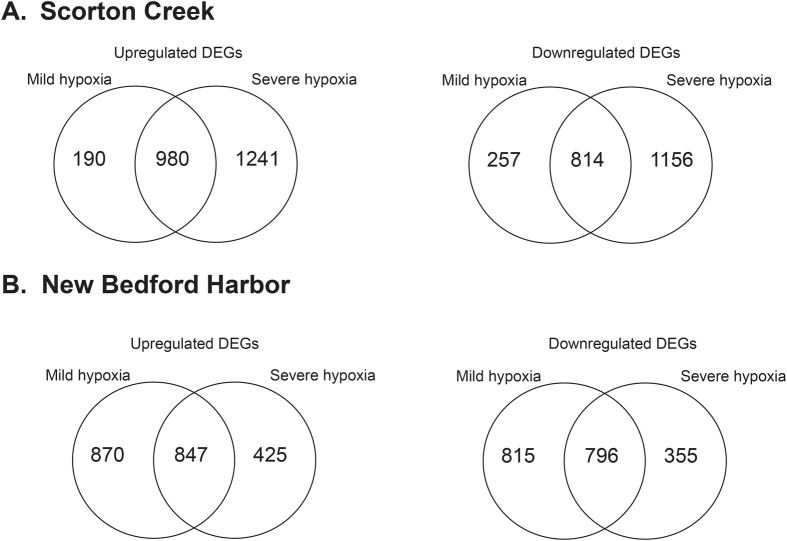
**Differentially expressed genes in response to hypoxia in NBH and SC fish.** Venn diagrams showing unique and common genes in response to mild and severe hypoxia in (A) SC fish and (B) NBH fish.

**Fig. 3.**
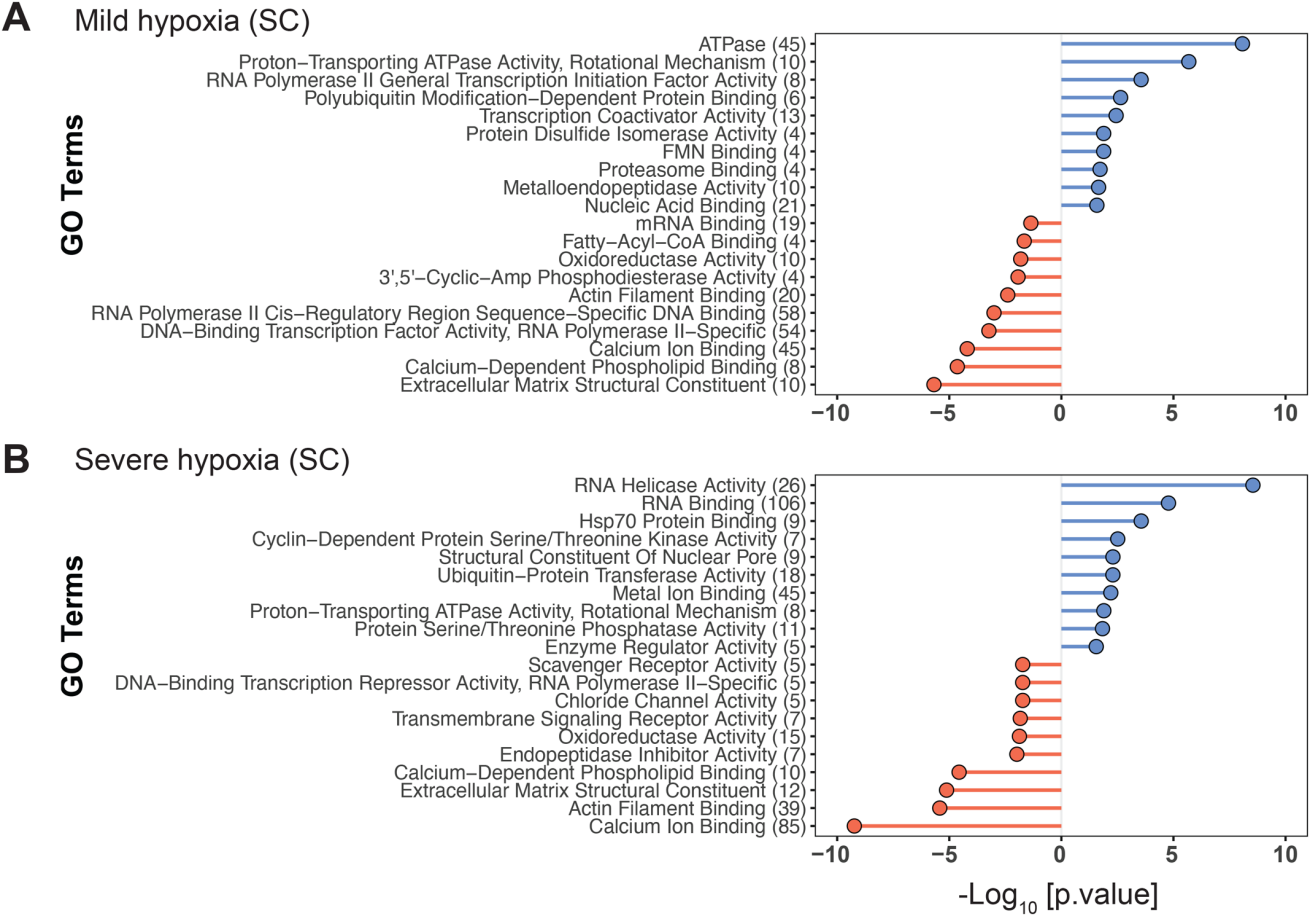
**Gene Ontology (Molecular Function) terms enriched among differentially expressed genes (DEGs) in mild hypoxia (A) and severe hypoxia (B) treatment groups in SC fish.** Only top 10 terms enriched among up- and downregulated genes are shown. Entire list of GO biological process and molecular function terms are provided in the supplementary information. The numbers in the parenthesis represent the number of DEGs represented in each GO term. Detailed description of filtering of GO terms to remove redundancy is described in Materials and Methods. GO terms enriched among upregulated DEGs are in blue and those from downregulated genes are in red.

We observed a dose-dependent effect of hypoxia on differential gene expression in SC fish. Exposure to mild hypoxia revealed 2241 differentially expressed genes (DEGs), with 1170 upregulated and 1071 downregulated. In response to severe hypoxia, 4191 DEGs were observed, with 2221 upregulated and 1970 downregulated. A total of 1794 DEGs were shared between the two hypoxia groups, with 980 upregulated and 814 downregulated genes (Fig. 2A). Gene Ontology (GO) analysis of DEGs from mild and severe hypoxia treatment groups revealed overrepresentation of GO molecular function (MF) terms related to ATPase activity, RNA binding, proteosome and extracellular matrix functions. The list of top 10 overrepresented GO:MF terms among up- and downregulated DEG in SC mild and severe hypoxia groups are shown in Fig. 3.

#### NBH

In NBH fish, exposure to mild and severe hypoxia elicited differential expression of 3328 and 2423 genes, respectively. Among the 3328 genes differentially expressed in response to mild hypoxia, 1717 were upregulated and 1611 genes were downregulated. Whereas in response to severe hypoxia, 1272 of the 2423 DEGs were upregulated, and 1151 genes were downregulated. Comparison of the upregulated DEGs from the mild and severe hypoxia groups revealed 847 genes shared between the two hypoxia groups (Fig. 2B), while a similar comparison of downregulated DEGs revealed 796 shared DEGs.

**Fig. 4.**
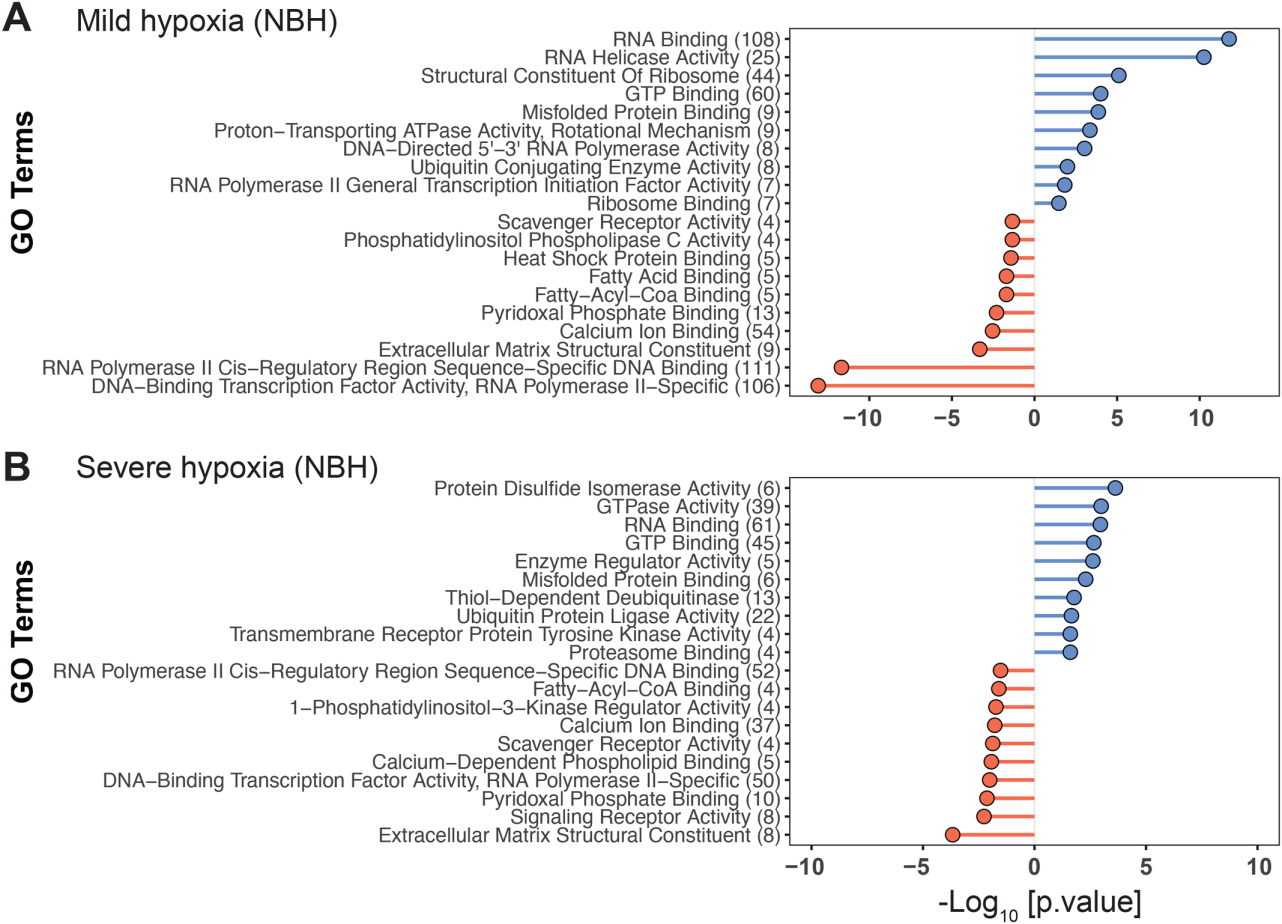
**Gene Ontology (Molecular Function) terms enriched among differentially expressed genes (DEGs) in mild hypoxia (A) and severe hypoxia (B) treatment groups in NBH fish.** Only top 10 terms enriched among up- and down-regulated genes are shown. Entire list of GO biological process and molecular function terms are provided in the supplementary information. The numbers in the parenthesis represent the number of DEGs represented in each GO term. Detailed description of filtering of GO terms to remove redundancy is described in Materials and Methods. GO terms enriched among upregulated DEGs are in blue and those from downregulated genes are in red.

GO analysis of mild and severe hypoxia upregulated DEGs showed overrepresentation of GO:MF terms related to mRNA splicing, translation, and proteasomal degradation. The terms enriched among downregulated DEGs include specific pathways such as cell adhesion and extracellular matrix functions. The top 10 overrepresented terms among up and downregulated DEGs are shown in Fig. 4.

#### Population differences

**Fig. 5.**
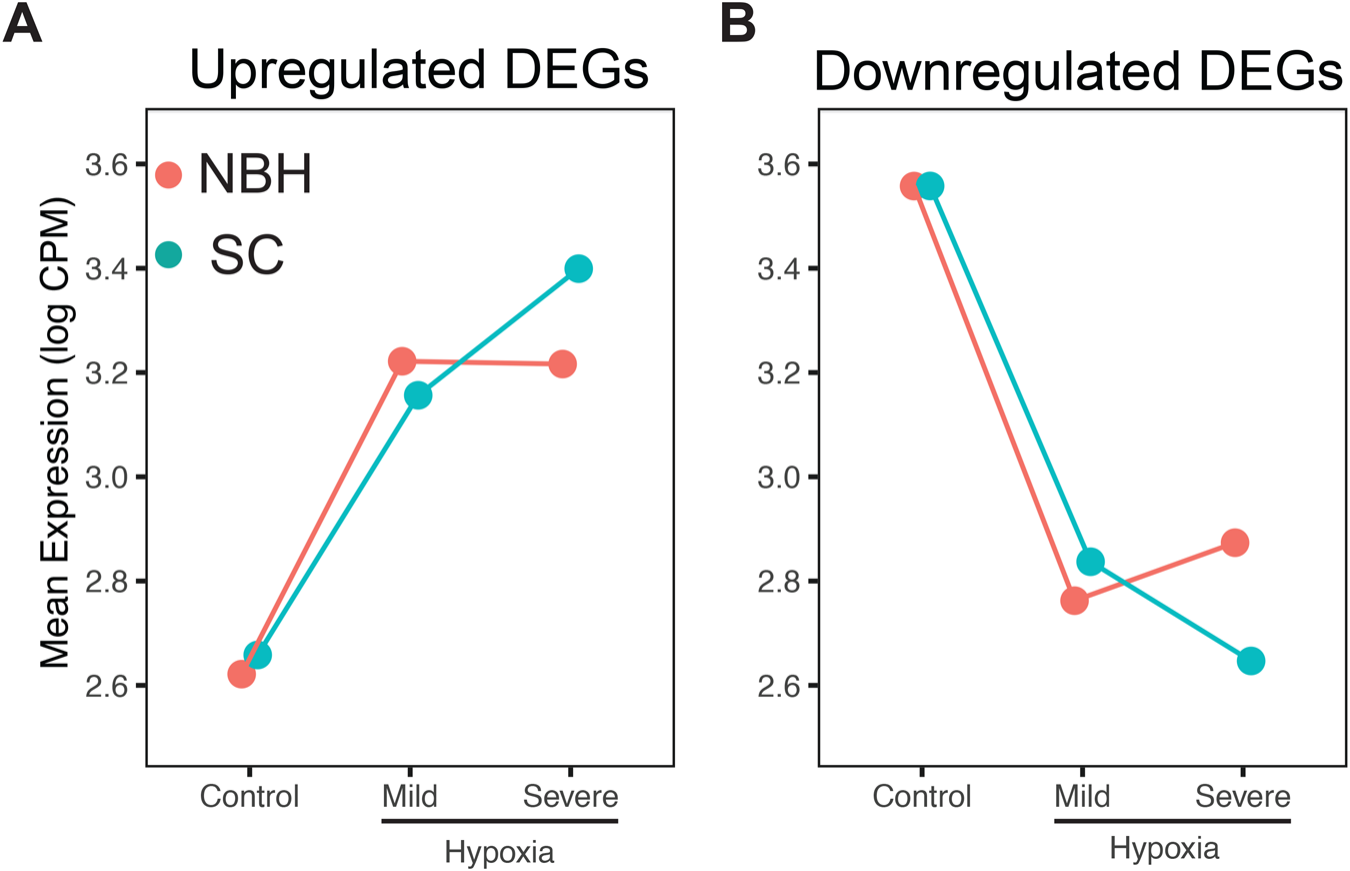
**Reaction norm plots showing gene expression patterns in response to two levels of hypoxia in NBH and SC fish.** Mean expression (Log counts per million (cpm)) of all the differential expressed genes in response to mild and severe hypoxia were plotted for up- (A) and downregulated (B) genes in NBH and SC fish.

Comparison of NBH and SC control groups revealed differential expression of 307 genes. Among them 159 and 148 are up- and downregulated in NBH, respectively, in comparison to SC. The GO terms enriched among these genes are shown in Fig. S3. Comparison of mean expression (log counts per million) of all up- and downregulated DEGs pooled from both populations and hypoxia treatment showed a dose-dependent response in SC fish, whereas NBH fish have a more muted gene expression response to severe hypoxia (Fig. 5).

**Fig. 6.**
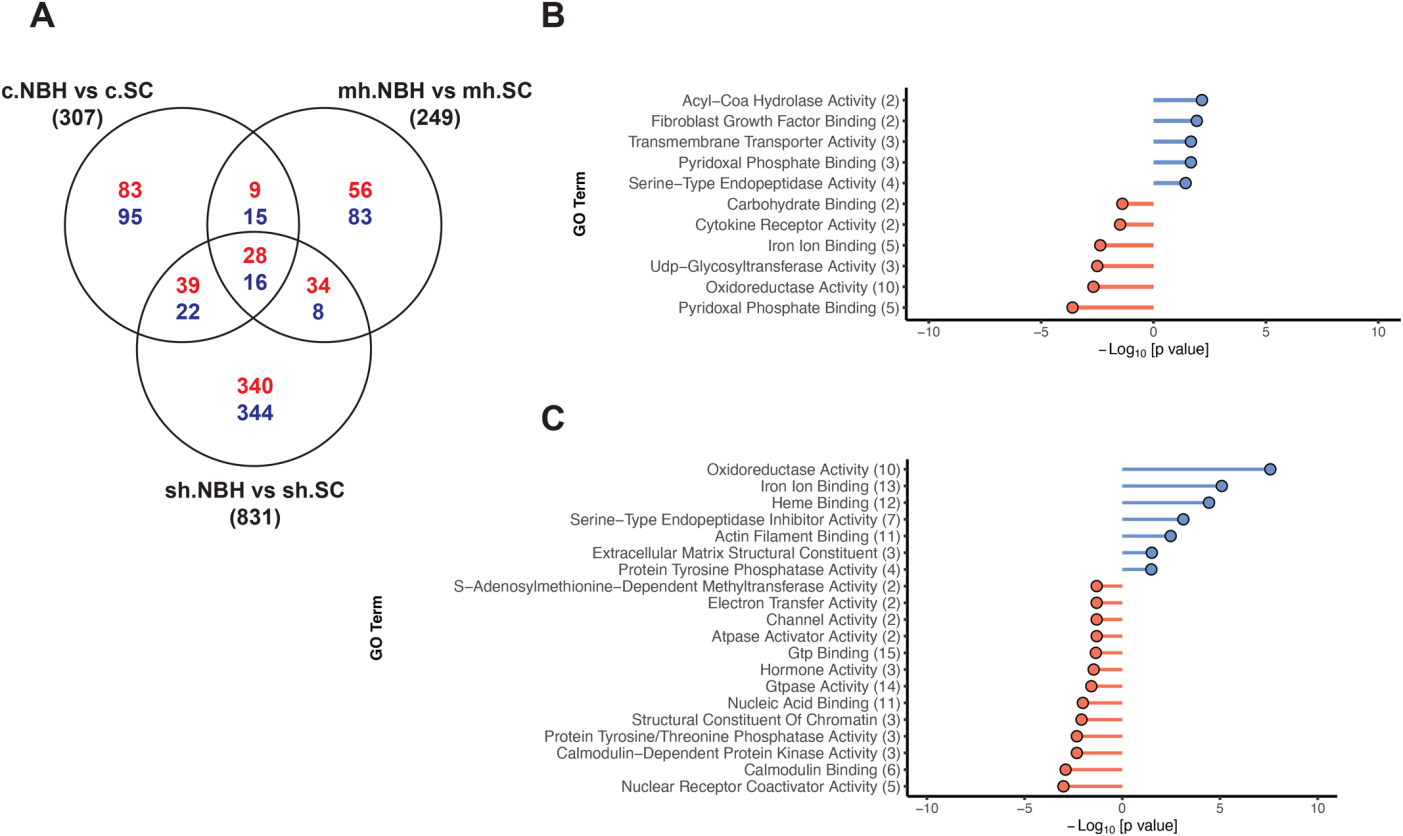
**Comparison of hypoxia treatment groups between populations.** (A) Venn diagram of differentially expressed genes (DEGs) in NBH fish in comparison to SC among control and hypoxia treatment groups. Up- and downregulated genes are in red and downregulated genes are in blue color. (B) Gene Ontology (molecular function) terms enriched among DEGs in NBH control group in comparison to SC control group and (C) Gene Ontology (molecular function) terms enriched among DEGs between NBH and SC hypoxia treatment groups. Due to small number of DEGs in mild hypoxia group, we pooled both mild and severe hypoxia groups for GO analysis. The complete list of GO biological process and molecular function terms are provided in the supplementary information. The numbers in the parenthesis represent the number of DEGs represented in each GO term. Detailed description of filtering of GO terms to remove redundancy is described in Materials and Methods. GO terms enriched among upregulated DEGs are in blue and those from downregulated genes are in red.

We compared hypoxia treatment groups between populations and identified 249 DEGs under mild hypoxia in NBH compared to SC. Of these, 127 genes were upregulated, and 122 were downregulated. Under severe hypoxia, there were 831 DEGs in NBH relative to SC, with 441 genes upregulated and 390 downregulated. We pooled the DEGs of mild and severe hypoxia for GO analysis mainly due to the small number DEGs between populations exposed to mild hypoxia. DEGs that were upregulated in NBH fish were enriched MF terms such as oxidoreductase activity, iron and heme binding, and downregulated genes were enriched in MF terms that included protein tyrosine kinase and phosphatase activity, nucleic acid and chromatin binding, and electron transport chain pathways (Fig. 6). The full list of DEGs and GO analysis results are available in the supplementary information. This analysis largely supports the findings observed within the population-level hypoxia treatment effects (described above), showing that NBH fish, on average, exhibit a muted transcriptional response to hypoxia compared to SC fish.

### Hypoxia-induced DNA methylation changes are pronounced in toxicant-resistant fish

Reduced representation bisulfite sequencing yielded 547.1 million total paired reads (14.4 to 33.3 million paired reads per sample). Of these reads, 544.5 million (99.5%) remained after quality-trimming. From the trimmed reads, 88.6% to 94.5% total reads were aligned to the *F. heteroclitus* genome. The detailed list of mapping statistics, raw and trimmed reads per sample is provided in the supplementary information (RRBS_Supplementary_information.xlsx). A total of 439,469 CpGs (5.4% of the 8,094,243 CpGs in the *F. heteroclitus* genome) had between 10× and 500× coverage in at least one sample after CpG filtering. Among them, 148,752 CpG sites (35.2% methylated and 64.8% unmethylated) were present in all samples and were used in downstream analysis.

**Fig. 7.**
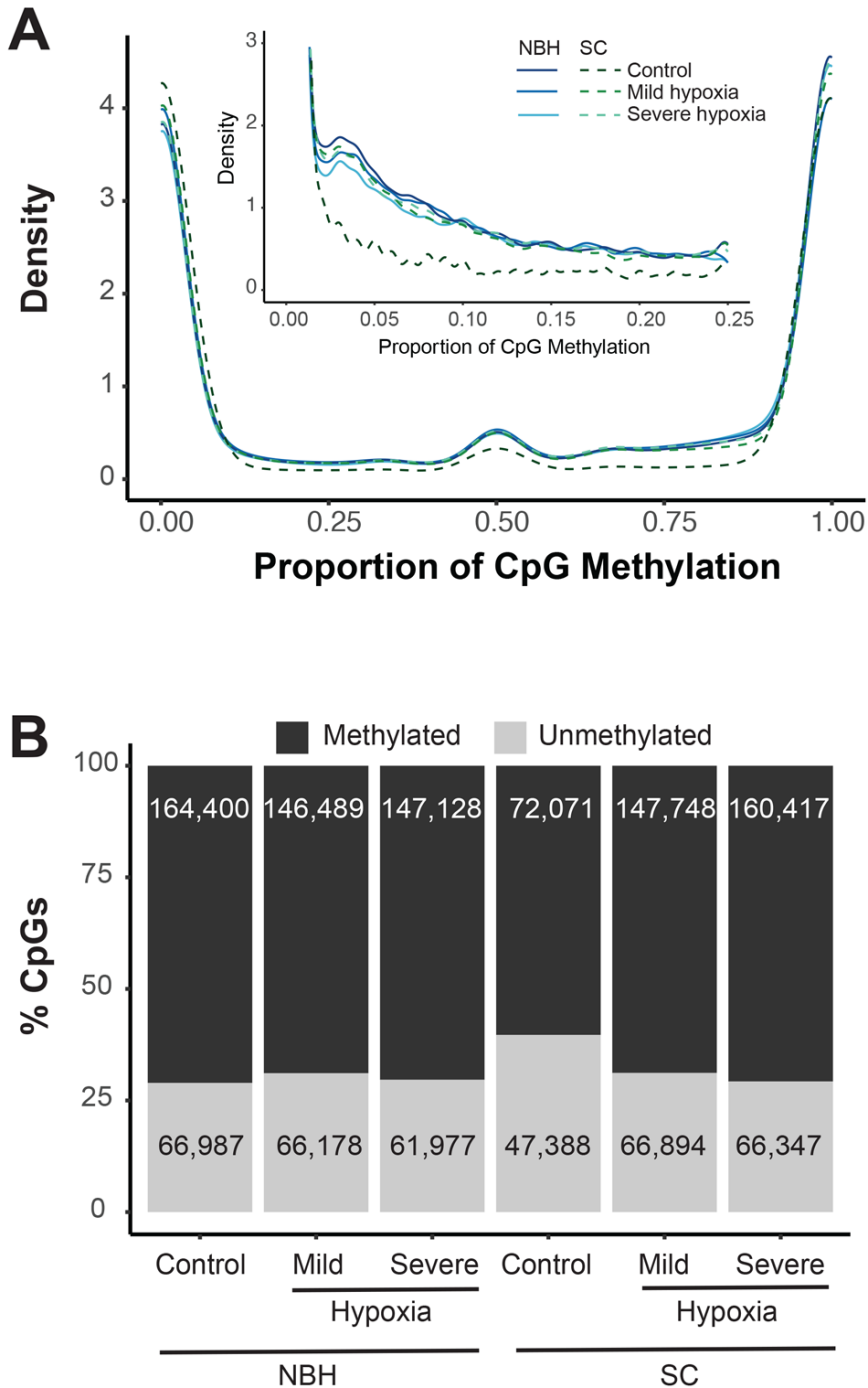
**DNA methylation landscape in *F. heteroclitus*.** (A) CpG DNA methylation density plots showing proportion of CpG methylation in different population and treatment groups. Inset shows the density plots of CpG sites with methylation levels below 25%. (B) Percent of methylated (>0% methylation; dark grey) and unmethylated (0% methylated; light grey) CpGs in various population and treatment groups. The average number of methylated and unmethylated CpGs are shown.

There was no statistically significant difference in global methylation level between NBH (28.2%) and SC liver samples (28.8%). Fig. 7 shows the mean CpG methylation density plots and total number of CpG sites in all the treatment groups. Only one DMR was identified between NBH and SC fish exposed to control conditions. This DMR is in chromosome 10 in an intron of SHISA6, a gene that encodes AMPA glutamate receptor subunit (Klassen et al., 2016). This DMR also overlaps with an annotated CpG island (identified as CpG island 77 in the genome) and is hypomethylated in NBH in comparison to SC fish.

**Fig. 8.**
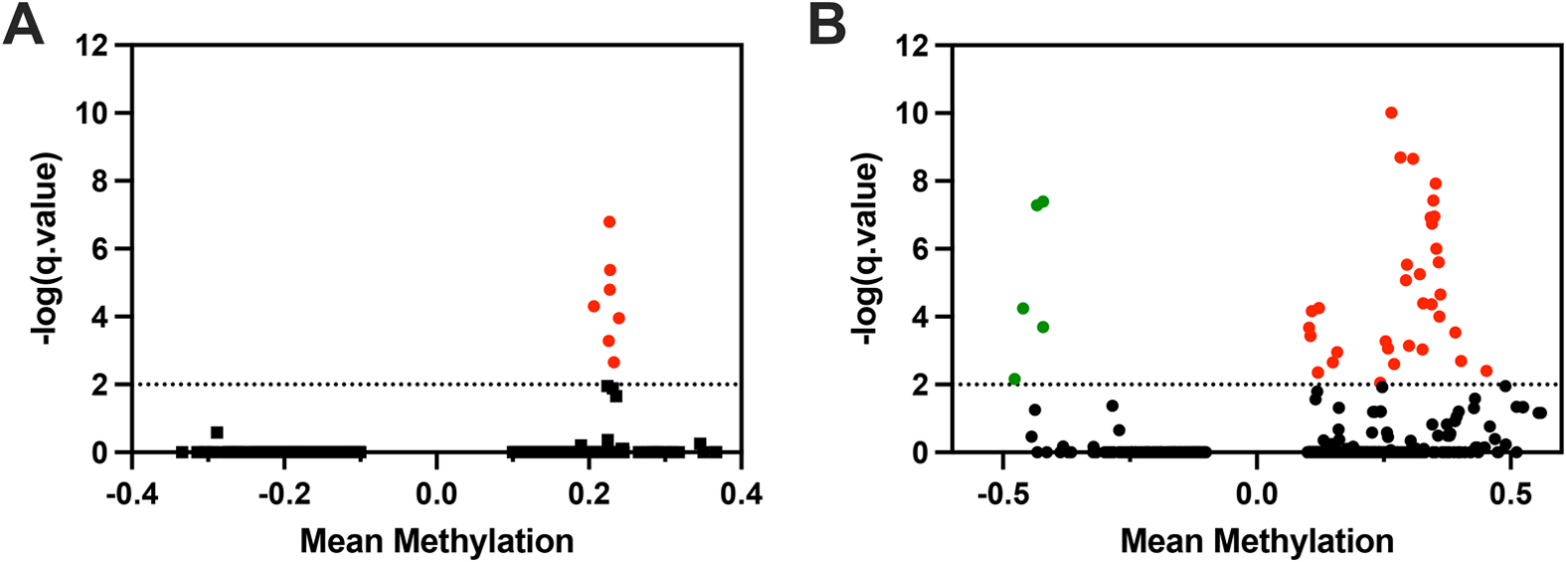
**Volcano plot showing differentially methylated regions (DMRs) in response to (A) 10% (mild) and (B) 5% (severe) hypoxia exposure in NBH fish.** Mean methylation difference (x-axis) between severe hypoxia and control group is plotted against q-value (y-axis). Each green and red spot represents a statistically significant hypo- and hypermethylated region, respectively.

Hypoxia exposure did not substantially alter global DNA methylation levels in both populations. In SC fish, global CpG DNA methylation levels were 28.8% in the controls and mild hypoxia, and 29.2% in severe hypoxia group. No DMR were observed in response to mild or severe hypoxia in this population. In NBH fish, global DNA methylation level was 27.9% in the control group, 27.2% in mild hypoxia, and 29.7% in severe hypoxia. Comparison of control and mild hypoxia groups revealed 10 DMR, all of which were hypermethylated. Similar comparison between control and severe hypoxia revealed 59 DMRs. Among them five were hypomethylated and 54 were hypermethylated (Fig. 8). The majority of the NBH DMR were found in CpG islands annotated in the genome. Only two DMR were shared between the mild and severe hypoxia groups, and they were hypermethylated. The genomic coordinates of these DMR are provided in supplementary information. We did not observe any significant correlation between DNA methylation in DMRs and the expression level of the associated genes (Figs S4 and S5)

## DISCUSSION

The findings from this study demonstrate a shift in physiological responses in fish adapted to toxicants and how this shift could compromise their capacity to respond to secondary stressors such as hypoxia. Our results show that both toxicant-sensitive (SC) and resistant killifish (NBH) respond to hypoxia but there are considerable differences in their responses as measured by gene expression and DNA methylation patterns. Atlantic killifish from SC show dose-dependent changes in gene expression patterns in response to hypoxia and no changes in DNA methylation patterns, whereas the fish from NBH showed a muted gene expression response to severe hypoxia suggesting compromised ability to mount a stress response. However, NBH fish showed a modest but significant DNA methylation changes in response to hypoxia. Together, these results reveal different response mechanisms and strategies to cope with hypoxia between toxicant-sensitive and toxicant-resistant fish populations.

### HIF signaling in toxicant-sensitive and toxicant-resistant populations

Transcriptional responses to hypoxia are well-documented in fish species (Qin et al., 2023; Shang et al., 2022a,b; Zhao et al., 2022; Feng et al., 2022; Beck et al., 2016), including Atlantic killifish (Flight et al., 2011; Townley et al., 2017; Thomas and Kinsey, 2024). Our findings demonstrate that both SC and NBH killifish exhibit responses to hypoxia, though their reactions differ significantly. The hypoxia-inducible transcription factors (HIF-1α, HIF-2α, HIF-3α) play a major role in the transcriptional activation of the hypoxic response (Lisy and Peet, 2008). The expression of these genes under hypoxic conditions depends on the intensity and duration of hypoxia exposure. Interestingly, we did not observe any changes in the expression of HIF1α and HIF2α genes in response to mild or severe hypoxia in either of the populations. However, we observed an increased expression of HIF3α only in NBH fish (mild hypoxia −log FC 1.75, FDR 7.45E-04; severe hypoxia −log FC 1.54, FDR 4.07E-03). A previous study in a closely related species, *Fundulus grandis*, showed no significant differences in mRNA expression of any of these HIF genes in response to hypoxia (1 mg oxygen l^−1^) after 6 and 24 h post-exposure (Murphy et al., 2023). However, studies in mammalian cell culture systems have shown differences in temporal profiles of HIF genes in response to hypoxia. In human endothelial cells, HIF-1α expression is maximal after 4 h of hypoxia and then is dramatically reduced by 8 h (Jaskiewicz et al., 2022a,b). In contrast, HIF-2α is maximal at 8 h and remains elevated up to 24 h (Jaskiewicz et al., 2022a,b; Moszynska et al., 2022). Unlike HIF-1α and -2α, moderate hypoxia exposure induced HIF3α mRNA expression within 2 h (Jaskiewicz et al., 2022a,b). However, very little is known about the functional role of HIF3α in hypoxia responses. The upregulation of HIF3α in response to hypoxia only in NBH fish suggests that adaptation to a contaminated environment may have altered its regulation, with potential implications for the hypoxia response. Further studies are needed to understand the roles of all three HIFs under different hypoxic conditions and at different life stages.

Another set of HIF pathway genes that were differentially expressed are the prolyl hydroxylase domain-containing proteins (PHD) (Fong and Takeda, 2008). There are three 2-oxoglutarate-dependent PHD proteins (PHD1, PHD2, and PHD3, encoded by *EGLN2*, *EGLN1*, and *EGLN3*, respectively) that are involved in HIFα ubiquitination and proteasomal degradation under normoxic conditions (Hoffman et al., 2001). Hypoxia has been shown to induce the expression of *EGLN1* and *-3* mRNAs, but not *EGLN2*, in several cell types (Ivan and Kaelin, 2017). In most cases, the induction of *EGLN3* mRNA is much more prominent than that of *EGLN1*. We observed a several-fold increase in the *egln3* expression (SC: log FC 8.77, FDR 1.82E-06; NBH: log FC 3.51, FDR 0.028) and modest but significant *elgn1a* upregulation in response to severe hypoxia in both populations (SC: log FC 1.33, FDR 9.59E-04; NBH: log FC 0.9, FDR 0.03). We also observed upregulation of *egln2* (logFC 1.33, FDR 9.59E-04) in response to severe hypoxia in SC fish, while it was not significant in NBH fish. Although very little is known about the role of *egln2* in oxygen sensing and hypoxia tolerance, the differences in expression between the two populations are intriguing. It has been suggested that increase in EGLN activity during hypoxia acts as a regulatory feedback loop for fast elimination of HIF after reoxygenation (del Peso et al., 2003).

In addition to the direct regulation of HIF transcription by hypoxia, multiple signaling pathways have been shown to play a role in the regulation of HIF gene expression in a variety of model systems. These include the PI3K-mTOR, interleukin-6 (IL-6), ERK, and MAPK signaling pathways (Luo et al., 2022). We observed differential expression of several genes associated with these signaling pathways only in NBH fish, suggesting a role in adaptation to toxicants. Notable among them include serine/threonine-protein kinase mTOR (*mtor*), phosphatidylinositol 4,5-bisphosphate 3-kinase catalytic subunit alpha (*pik3r1*) and RAC-beta serine/threonine-protein kinase (*akt2*). Despite the lack of an increase in HIF-1α expression, the differential expression of these signaling pathways suggests that NBH fish use crosstalk between multiple signaling pathways to cope with hypoxia in a way that appears to be distinct from SC fish. There is growing evidence that HIF-, PI3K-mTOR and ERK-MAPK signaling pathways act in an integrated way, influencing downstream pathways that affect cellular metabolism and survival during hypoxia (Luo et al., 2022). The differential expression of genes involved in the mTOR signaling pathway suggests disruptions in protein folding and secretion, triggering the unfolded protein response (UPR), which aids in restoring endoplasmic reticulum homeostasis (Wouters and Koritzinsky, 2008). The role of UPR in oxidative stress and hypoxia is well-established (Guan et al., 2024) and toxicant-resistant NBH fish would be an ideal model to investigate the effects of chronic exposure to toxicants on protein endoplasmic reticulum (ER) stress.

### Transcriptional responses to hypoxia

While responses to hypoxia are well documented across various organisms, encompassing metabolic changes, angiogenesis, and vascularization (Borowiec et al., 2018, 2015; Borowiec and Scott, 2020; Luo et al., 2022), our results, based on a 6-h hypoxia exposure, revealed gene expression changes that were more focused on transcription, translation, and cell cycle-related genes, rather than the classical hypoxia responses. This contrasts with studies in *in vitro* mammalian systems, where hypoxia target genes are differentially expressed in shorter timescales (Wu and Yotnda, 2011). The lack of similar responses in our study could be due to differences in the timescales of transcription and translation between fish maintained at 20°C and mammalian cells held at 37°C (Wu and Yotnda, 2011; Wenger et al., 2015).

We analyzed population differences in response to hypoxia using two approaches: first, by comparing hypoxia responses within each population, and second, by comparing hypoxia treatments between populations. Both approaches yielded similar conclusions. SC fish exhibited dose-dependent changes in gene expression, indicating a gradual and proportional response to varying levels of hypoxia. In contrast, NBH fish demonstrated drastic gene expression changes in response to mild hypoxia, but a muted response to severe hypoxia. This pattern suggests that NBH fish have a lower tolerance threshold for hypoxic conditions and are unable to induce a robust transcriptional response to severe hypoxia. This could be potentially due to higher energetic costs for survival in contaminated environments, leading to compromised ability to respond to additional stressors (Jayasundara et al., 2017; Jasperse et al., 2023).

In SC fish, mild hypoxia exposure caused an overrepresentation of genes associated with efflux transporters, transcription factor activity, mRNA and nucleic acid binding, and proteasomal functions. All these functions have been previously shown to be altered by hypoxia (Kallio et al., 1999; Vadlapatla et al., 2013; Thews et al., 2008; Yfantis et al., 2023; Chee et al., 2019). For instance, efflux pumps belonging to the ATP-binding cassette (ABC) superfamily of membrane transporters are known to play a significant role in cellular protection against oxidative stress (Patak et al., 2011). Several genes within the ABC family of efflux transporters are expressed in the liver and are involved in the transport of glutathione and glucuronide conjugates (Wlcek and Stieger, 2014; Kipp and Arias, 2002; Chan and Vandeberg, 2012). These findings suggest that mild hypoxia increases reactive oxygen species (ROS), with glutathione playing a crucial role in neutralizing free radicals and protecting the liver. The glutathione conjugates are subsequently eliminated from hepatocytes by the efflux pumps, contributing to cellular detoxification (Chan and Vandeberg, 2012). Additionally, mild hypoxia caused the upregulation of genes associated with RNA polymerase II (RNAPII) general transcription factor activity and the downregulation of cis-regulatory sequence-specific DNA binding RNAPII activity. This is not surprising, given the evidence that hypoxia affects the transcription of hundreds of genes (Kindrick and Mole, 2020). Under normoxic conditions, almost all HIF target genes display an open chromatin structure and harbor transcriptionally active but paused RNAPII (Soliman et al., 2024). Changes in the expression of RNAPII activity genes in response to hypoxia suggests the release of paused polymerase II into productive RNA synthesis by recruiting various coactivators, repressors, and chromatin remodelers, resulting in either the activation or inhibition of transcription of target genes (Soliman et al., 2024).

In response to severe hypoxia, SC fish showed an overrepresentation of genes associated with RNA helicases. They play an important role in cellular RNA metabolism, including transcription, pre-mRNA splicing, RNA export, storage, decay, and translation. Recently, RNA helicases have been shown to be involved in many biological processes, including DNA damage repair, cellular stress response, hypoxia and antiviral defense (Wang et al., 2021; Cai et al., 2017). Another major overrepresented group of genes in response to severe hypoxia in SC fish are the RNA binding proteins (RBPs) – important players in mRNA turnover (decay and stabilization) and translation (Mitchell and Tollervey, 2001). RNA helicases and RBPs are essential for the adaptive cellular response to hypoxia (Wang et al., 2021; Masuda et al., 2009). While there is very little understanding of the role of RBPs in environmental model species such as killifish, the upregulation of these genes in response to hypoxia suggests highly conserved cellular mechanisms.

Interestingly, the genes and pathways that were upregulated in SC fish under severe hypoxia were similarly upregulated in NBH fish exposed to mild hypoxia. This suggests that the toxicant-adapted NBH fish are more sensitive to mild hypoxia, undergoing more drastic transcriptional changes. The upregulation of RNA helicases and RBPs indicates post-transcriptional regulation of pre-existing RNAs (Masuda et al., 2009), suggesting that NBH fish may reduce transcription under stress. Indeed, this was observed under severe hypoxia in NBH fish, where the number of DEGs was lower than it was in SC fish, indicating a drastic reduction in transcription.

Hypoxia exposure downregulated genes related to calcium signaling, oxidoreductase activity, and extracellular matrix (ECM) modeling proteins. These pathways were altered in both hypoxia treatments and across both populations, suggesting they are vital for cellular response to hypoxia. It is well established that hypoxia impairs mitochondrial respiration and ATP synthesis, leading to an increased production of reactive oxygen species (ROS) and calcium release from the endoplasmic reticulum into the cytosol (Luo et al., 2022). The resulting elevated cytosolic calcium levels cause increased calcium uptake into mitochondria and mitochondrial calcium overload, which in turn leads to mitochondrial depolarization and the initiation of cell death (Seta et al., 2004). The decreased expression of genes associated with calcium signaling suggests an adaptation toward hypoxia tolerance. Similarly, hypoxia involves a switch from oxidative phosphorylation to glycolysis, resulting in increased production of NADH and an imbalance in the NAD+ and NADH ratio, causing altered redox potential (Li et al., 2023; Janczy-Cempa et al., 2022). These conditions favor the overexpression of many redox enzymes, such as cytochrome P450 reductase and nitroreductases. Even though these genes were not altered, genes that are dependent on NAD+ or NADH and play critical roles in metabolism were downregulated. They include *agmo* (alkylglycerol monooxygenase), *sc5d* (sterol-C5-desaturase), *hsd11b2* (11-β-hydroxysteroid dehydrogenase 2), *bdh1* (d-beta-hydroxybutyrate dehydrogenase), and *foxred1* (FAD-dependent oxidoreductase domain-containing 1) (Li et al., 2023). It remains to be determined whether the downregulation of these genes under hypoxia is adaptive or maladaptive.

Another significant group of genes that are commonly downregulated in response to hypoxia are ECM components genes. The ECM is a complex network of proteins and other molecules that provide structural and biochemical support to surrounding cells (Naba et al., 2012). Its composition and function are crucial for tissue integrity, cell behavior, and overall organism health (Gilkes et al., 2014). Hypoxia has been shown to cause significant effects on the ECM in various aquatic species (Mu et al., 2020; Rojas et al., 2024; Shang et al., 2022a,b; Chen et al., 2021). In addition, there is growing evidence suggesting the role of hypoxia and HIFs in reprogramming cancer cells by regulating extracellular matrix (ECM) deposition, remodeling and degradation, thereby promoting cancer metastasis (Bertout et al., 2008). We observed downregulation genes associated with collagen synthesis, which maintain the structural integrity of tissues. This could lead to weakened tissue architecture or cause tissue remodeling such as changes in vascularization, which can be adaptive to survive under hypoxic conditions. The molecular mechanisms associated with these changes could be either by direct regulation of ECM pathway genes by HIF proteins or indirectly by the induction of oxidative stress (Gilkes et al., 2014; Vujić et al., 2022). Overall, the effects of hypoxia on the extracellular ECM in killifish highlight the importance of this adaptive function in coastal fish species, which frequently encounter periodic hypoxic conditions.

### ARNT as a limiting factor in toxicant-resistant NBH fish

ARNT serves as a shared dimerization partner for both the AhR and HIF-1α signaling pathways, raising the possibility of competition between these pathways for ARNT. This hypothesis has been explored primarily using *in vitro* models, which demonstrate that ARNT can act as a limiting factor (Vorrink and Domann, 2014; Chan et al., 1999; Zhang et al., 2022). For instance, activation of the hypoxia pathway has been shown to inhibit AHR agonist-induced *cyp1a* upregulation, while activation of the AHR pathway has been shown to have either an additive effect (Chan et al., 1999) or no inhibitory effect on the expression of hypoxia responsive genes (Zhang et al., 2022). Similar patterns have been observed in other systems (Nie et al., 2001).

In this study, NBH fish exhibit constitutively high *cyp1a* expression compared to SC fish (log FC 1.306, FDR 0.009), suggesting that parental exposure to dioxin-like PCBs had a cross-generational effect on gene expression patterns in laboratory reared offspring. If ARNT is indeed limiting, NBH fish would be expected to show a reduced capacity for hypoxia responses. Consistent with this, we observed a muted response to severe hypoxia in NBH fish, suggesting that ARNT availability may be constrained. However, further studies are required to elucidate AHR:ARNT and HIF:ARNT protein interactions in these populations to confirm this hypothesis. Basal expression levels of ARNT genes (*arnt* and *arnt2*) do not differ significantly between the two populations, though *arnt* is differentially expressed in response to hypoxia in both. These findings suggest that assessing ARNT interactions at the protein level will provide stronger evidence for its role as a limiting factor and its influence on environmental adaptation in NBH fish.

### Epigenetic changes in responses to hypoxia

Epigenetic effects, particularly DNA methylation changes in response to hypoxia, are well documented across a variety of fish species (Ragsdale et al., 2022; Thoral et al., 2022; Gu et al., 2024; Jones and Griffitt, 2022; Kelly et al., 2020). Hypoxia has been shown to cause both global and gene-specific alterations in DNA methylation patterns, which can influence gene expression, development, and stress response pathways. For instance, in species like zebrafish (*Danio rerio*) and Atlantic salmon (*Salmo salar*), hypoxic conditions have been associated with both hypermethylation and hypomethylation of key regulatory genes involved in metabolic adaptation and oxidative stress responses (Ragsdale et al., 2022; Kelly et al., 2020). These epigenetic changes are hypothesized to help fish cope with hypoxia by modulating critical pathways for survival, growth, and development.

To our knowledge, this is the first study where genome-wide DNA methylation patterns are profiled in Atlantic killifish. In this study, we hypothesized that resistant and sensitive populations of Atlantic killifish would exhibit distinct hepatic DNA methylation patterns, possibly reflecting their differential tolerance to dioxin-like PCBs. However, we did not observe distinct population differences in DNA methylation (NBH versus SC control groups), suggesting that parental exposure to dioxin-like PCBs did not have a detectable effect on DNA methylation in the offspring held under normoxic conditions.

Hypoxia exposure induced DNA methylation changes that were markedly different in the two populations. In the sensitive SC population, no differentially methylated regions (DMRs) were identified in response to either mild or severe hypoxia. In contrast, hypoxia resulted in significant changes in DNA methylation in the toxicant-resistant NBH population. Most of these DMRs are located in intronic regions and were enriched within CpG islands, which are known to play a critical role in regulating gene expression (Deaton and Bird, 2011). These findings suggest that the NBH population may have a more plastic epigenetic response to hypoxia compared to the SC population, which could reflect underlying differences in their adaptive capacities. While further research is needed to elucidate the functional consequences of these methylation changes, this study provides new insights into the epigenetic mechanisms by which fish populations respond to environmental stressors like hypoxia.

The majority of hypoxia induced DMRs are hypermethylated, supporting previous observations that hypoxia cause DNA hypermethylation (Thienpont et al., 2016; Rosa Ng and Jain, 2016; Robinson et al., 2012). Associating DMRs with genes revealed that some of the DMRs are related to genes involved in sialylation, vascularization and development. Four DMRs were associated with the gene *St6galnac3*, which is involved in the sialylation pathway. Sialylation refers to the addition of sialic acid units to oligosaccharides and glycoproteins (Li and Ding, 2019). Sialic acids moieties act as bridging molecules, facilitating communication between cells and the extracellular matrix. Additionally, one DMR was associated with *beta-chimaerin*, a protein linked to vascularization (Ben Dhaou et al., 2022). Hypoxia has been shown to influence both sialylation and vascularization processes, particularly in cancer models (Ben Dhaou et al., 2022; Jones et al., 2018), suggesting that similar mechanisms may be involved in the hypoxic responses observed in this study. However, we did not observe any significant correlation between these DNA methylation changes and expression of the associated genes. None of the 58 genes associated with DMRs in NBH fish were differentially expressed suggesting a temporal lag in DNA methylation changes and gene expression. In addition, several studies have shown that the majority of the DNA methylation changes are not correlated with gene expression (Aluru et al., 2018; Lindner et al., 2021; Bogan and Yi, 2024). This suggests that epigenetic regulation of gene expression is multilayered, with many levels of control, involving DNA methylation, histone modifications and chromatin organization.

While differential methylation did not correlate with gene expression changes in NBH, we observed differential expression of several chromatin modifier genes, particularly histone lysine demethylases (KDMs), in response to hypoxia in both fish populations. This is expected, as KDMs belong to the family of 2-oxoglutarate-dependent dioxygenases, which function as oxygen sensors (Kindrick and Mole, 2020). The differentially expressed KDM genes include *kdm1aa*, *kdm2aa*, *kdm2ab*, *kdm2ba*, *kdm3b*, *kdm4b*, *kdm5ba*, *kdm5bb*, *kdm5c*, *kdm6a*, *kdm6ba*, and *kdm7aa*. These genes have been shown to be directly regulated by hypoxia-inducible factors (HIFs), linking their expression to the cellular response to hypoxic stress (Salminen et al., 2016; Lee et al., 2014). Given the differential expression of histone lysine demethylases in response to hypoxia, it would be intriguing to investigate the role of chromatin modifiers in the adaptation to hypoxia in environmental species, as they may be key regulators of gene expression in low-oxygen environments.

### Conclusions

This study highlights the complex and distinct physiological and epigenetic responses of Atlantic killifish populations adapted to toxicants when exposed to hypoxia. Our findings suggest that the capacity to respond to secondary stressors such as hypoxia may be altered in populations adapted to environmental contaminants. As expected, the toxicant-sensitive SC fish displayed a dose-dependent response to hypoxia exposure. However, the toxicant-resistant NBH fish, exhibited muted transcriptional responses but a more pronounced DNA methylation response to severe hypoxia, suggesting different molecular mechanisms in this population. Importantly, the differential DNA methylation patterns in response to hypoxia between the two populations indicate differences in epigenetic plasticity, which needs further investigation. A limitation of this study is the focus on liver tissue, which may not fully represent systemic responses to hypoxia. Additionally, physiological differences between the NBH and SC populations, shaped by their distinct habitats, may confound the interpretation of molecular responses. Overall, this research provides valuable insights into the diverse molecular mechanisms by which fish populations, with different environmental histories, respond to hypoxia and highlights the need for further exploration of epigenetic and chromatin-level responses in the context of environmental adaptation.

## MATERIALS AND METHODS

### Experimental fish

The animal husbandry and experimental procedures used in this study were approved by the Animal Care and Use Committee of the Woods Hole Oceanographic Institution. Mature adult male and female killifish from Scorton Creek (SC; Sandwich, MA, USA) and New Bedford Harbor (NBH; New Bedford, MA, USA) were collected during low tide in the month of June 2018 using minnow traps, as described previously (Karchner et al., 1999). The water temperature and salinity at the time of collection in NBH was 27°C and 34 parts per thousand (ppt) and in SC was 25°C and 33 ppt respectively. In NBH, fish were collected from the banks of the Acushnet River estuary (latitude 41.670, longitude −70.915) during receding tide. Fish from SC were collected from tidal pools (latitude 41.746, longitude −70.428). Fish were brought to the lab and maintained in the Redfield Laboratory (WHOI) with continuous flow-through seawater (SW) at 18–20°C, saturated dissolved oxygen (21% oxygen saturation or 7.21 mg oxygen l^−1^), and 14 h:10 h light:dark photoperiod conditions.

F1 generation of embryos from SC and NBH were obtained by *in vitro* fertilization following established protocols (Karchner et al., 1999). Briefly, four or five female fish from SC or NBH (15.5±1.2 g mean wet mass) were lightly anesthetized with Tricaine (MS222; buffered with sodium bicarbonate, Sigma-Aldrich, St. Louis, MO, USA) and oocytes were obtained for *in vitro* fertilization by gently squeezing the abdomen. Oocytes were collected in glass Petri dishes with filtered SW (30 parts per thousand; ppt). Milt was obtained by euthanizing 2-3 mature males (13.5±1.1 g mean wet mass) from the same population in MS222, dissecting out the gonads, and chopping them with a scalpel blade in seawater. A few drops of milt were added to the oocytes for fertilization. Approximately 20 min after the addition of milt, embryos were rinsed with filtered SW to remove any excess sperm. Fertilized embryos were reared at 23°C under 14 h:10 h light:dark photoperiod conditions until hatching. Larvae were raised in 2-gallon aquarium tanks in aerated seawater for 6 months. During larval rearing, fish were fed brine shrimp daily and water was exchanged every 2-3 weeks. Oxygen concentration was measured in the tanks once every 2-3 days and oxygen saturation was above 20% throughout the rearing period.

### Confirmation of toxicant resistant phenotype using quantitative real-time PCR (qRT-PCR)

A subset of F1 generation embryos from NBH and SC were exposed to either DMSO (0.01%; vehicle control) or 50 nM PCB126 from 4 h post-fertilization to 5 days post-fertilization through water borne exposure. Exposures were conducted in glass Petri dishes in 50 ml of seawater at 23°C under 14:10 h light:dark photoperiod regimen. Each treatment condition consisted of 30 embryos. At the end of the exposure, pools of six embryos were flash frozen in liquid nitrogen for RNA isolation (five replicates per treatment). Total RNA was isolated using the Aurum total RNA fatty and fibrous tissue kit (BioRad, Herculus, CA, USA) with in column DNase treatment. cDNA was prepared with using iScript cDNA synthesis kit (BioRad, CA, USA) and used in qRT-PCR to quantify *cyp1a* expression. *β-actin* was used as a housekeeping gene. The qRT-PCR primers used to amplify *cyp1a* were 5′-CTTTCACAATCCCACACTGCTC-3′ (forward primer) and 5′-GGTCTTTCCAGAGCTCTGGG -3′ (reverse primer). *β-actin* primers used were 5′-TGGAGAAGAGCTACGAGCTCC-3′ (forward primer) and 5′-CCGCAGGACTCCATTCCGAG-3′ (reverse primer). The PCR conditions used were 95°C for 3 min and 95°C for 15 s/66°C (*cyp1a* and *β-actin*) for 1 min (40 cycles). At the end of each PCR run, a melt curve analysis was performed to ensure that only a single product was amplified. Three technical replicates were used for each sample. Relative expression was normalized to that of *β-actin* {2^−ΔCt^; where ΔCt=[Ct(*cyp1a*)−Ct(*β-actin*)]}. *Cyp1a* mRNA expression levels in response to PCB126 exposure were compared to respective population controls using one-way ANOVA (GraphPad Prism version 5.3). A probability level of *P*<0.01 was considered statistically significant. The embryos from this clutch were reared for 6 months and used in hypoxia exposure experiment described below.

### Hypoxia exposure

Six-month-old killifish juveniles from SC (149±43 mg mean wet mass) and NBH (145±39 mg mean wet mass) were exposed to either mild (10% oxygen saturation, 3.46 mg O_2_ l^−1^; *n*=5 per population) or severe hypoxia (5% oxygen saturation, 1.72 mg O_2_ l^−1^ ; *n*=5 per population) for 6 h. These two hypoxia levels were chosen based on preliminary experiments with the same cohort of fish, where the loss of equilibrium (LOE) was assessed under 1% and 5% oxygen saturation in both NBH and SC fish. Six hours of exposure to 5% oxygen saturation did not cause LOE in fish from either population (*n*=5 individual fish per population), whereas 1% oxygen saturation caused LOE within 6 h in fish from both populations. The details of the results from the preliminary experiments are provided in Fig. S1.

Hypoxia exposure set up included Pyrex glass dishes (270 ml volume) equipped with oxygen sensor spots (PreSens Precision Sensing GmbH, Germany) placed inside hypoxia chambers (Stemcell Technologies) with pre-mixed air set to 5 or 10% oxygen pumped into the chambers continuously. A control group (normoxia, 20.9% oxygen saturation; *n*=5 per population) was maintained on the benchtop (Fig. 1). Prior to introducing the fish to hypoxia, 250 ml filtered seawater was added to the Pyrex glass dishes and was allowed to equilibrate overnight to ensure that the water had reached the respective treatment conditions. Oxygen levels in individual beakers were checked prior to introducing the fish using a FireString oxygen sensor (PyroScience, Germany) and were found to be at the treatment conditions in each of the individual dishes. At the start of the experiment, individual fish were quickly introduced into the Pyrex dishes and chambers closed immediately. Fish were maintained at treatment conditions for 6 h. At the end of the exposure period, oxygen levels were measured. Fish were rapidly anesthetized using a lethal dose of MS222 buffered with sodium bicarbonate. Livers were dissected and stored at −80°C until further analysis. Liver tissue was selected to quantify hypoxia-induced transcriptional and DNA methylation responses due to its central role in metabolism. Hypoxia has been widely shown to significantly impact metabolic pathways, and in toxicant-resistant killifish, studying the response to hypoxia offers valuable insights into how adaptation to toxicants influences metabolic homeostasis under low-oxygen conditions.

### Isolation of total RNA and genomic DNA from liver samples

Simultaneous isolation of genomic DNA and total RNA from liver tissues was performed using the ZR-Duet DNA/RNA Mini Prep kit (Zymo Research, CA, USA). RNA was treated with DNase during the isolation process. DNA and RNA were quantified using the Nanodrop Spectrophotometer. The quality of DNA and RNA was checked using the Agilent 4200 and 2200 Tape Station systems, respectively. The DNA and RNA integrity numbers of all samples were between 9 and 10.

### RNA sequencing

Libraries were constructed using Illumina stranded library preparation kit following manufacturer's protocol. Single end 50 bp reads were sequenced using Illumina HiSeq2500 platform. RNA sequencing library construction and sequencing were done at the Tufts University core facility.

### Reduced representation bisulfite sequencing (RRBS)

Library preparation was performed using the Premium RRBS kit (Diagenode). In brief, 100 ng DNA from each sample were enzymatically digested by the restriction enzyme MspI at 37°C for 12 h. Following ends preparation, a different set of adaptors was added to each sample and adaptor ligation was performed by the addition of ligase. Size selection of adaptor-ligated DNA fragments was performed by Agencourt AMPure XP beads (Beckman Coulter) and the DNA was eluted in Resuspension buffer (Diagenode, catalog number C02030033). Part of the eluted sample was subjected to qPCR using 2× KAPA HiFi HotStart ReadyMix (Kapa Biosystems) for quantification and subsequent pooling per nine samples. The pooling was performed according to two parameters: the Ct value and the adaptor ID of each sample. The pooling was followed by a cleanup with AMPure XP beads to reduce the volumes. Bisulfite treatment was performed, and bisulfite-converted DNA was eluted twice in BS Elution buffer (Diagenode, catalog number C02030033). Part of the bisulfite converted library was used in qPCR for the determination of the optimal cycle number for the enrichment PCR. 2× MethylTaq Plus Master Mix was used for the amplification PCR and a last cleanup with AMPure XP beads followed. PCR product was run on an 2% agarose gel to remove adaptor dimers. The quality of the final libraries was checked on an Agilent 2100 High Sensitivity DNA chip. The concentration was determined by performing qPCR on the samples using a dilution of PhiX index3 as standard. Paired end 50 bp reads were sequenced on an Illumina HiSeq4000 platform by a commercial facility (NXT-Dx, Ghent, Belgium).

### Genome information and feature tracks

The *F. heteroclitus* genome (https://www.ncbi.nlm.nih.gov/datasets/genome/GCF_011125445.2/) was used for all analyses. Genome feature information was pulled directly from the genome to generate gene, coding sequence (CDS), exon, and lncRNA genome feature tracks using Gnomom, RefSeq, cmsearch, and tRNAscan-SE annotations. Chromosome name and length information was also extracted from the genome to generate additional feature tracks using bedtools version 2.31.1 (Quinlan and Hall, 2010). A non-coding sequence track was created by using the complement of the CDS track (complementBed). Similarly, the intergenic genome feature track was created using the complement of the gene track. The intersection (intersectBed) between the non-coding sequence and gene tracks were used to create an intron track. All genome feature tracks are available in an Open Science Framework repository (doi.org/10.17605/OSF.IO/NZRA8).

### RNA sequencing and analysis

Raw data files were assessed for quality using FastQC version 0.11.9 (Andrews, 2010) prior to preprocessing. Preprocessing was done by trimming the adaptor sequences using Trimmomatic (version 0.25) and removing any reads with low sequence quality (Phred score <20) (Bolger et al., 2014). Trimmed sequence reads were mapped to the *F. heteroclitus* genome using the STAR aligner version 2.6.1d (Dobin and Gingeras, 2015). The number of reads mapped to annotated regions of the genome was obtained using HTSeq-count version 0.11.1 (Anders et al., 2015). Statistical analysis was conducted using edgeR version 3.40.2, a Bioconductor package (Robinson et al., 2010). Transcripts from all samples were compiled into a DGEList, and lowly expressed transcripts were filtered out using the filterByExpr function. Sample ordination was visualized using multidimensional scaling analysis with the ape version 5.8 package (Oksanen et al., 2022), revealing an outlying sample that was removed from subsequent analysis (Fig. S2). We used the quasi-likelihood model in edgeR (glmQLFTest) to perform differential gene expression analysis. Only genes with false discovery rate (FDR) of <5% were considered to be differentially expressed. Raw data has been deposited in gene expression omnibus (accession number GSE278569).

Functional annotation of DEGs was done using gene ontology (GO) biological process (GO:BP) and molecular function (GO:MF) terms. Identification of overrepresented GO terms (*P* value <0.05) among sets of DEGs was done using the enricher function in ClusterProfiler version 4.6.2 (Wu et al., 2021). The background gene list included all expressed transcripts in the filtered DGEList. Similar GO:BP or GO:MF terms were clustered based on the frequency of shared genes using the R package *rrvgo* 1.16.0 (Sayols, 2023). Representative parent terms from each cluster were chosen based on the lowest *P* value.

### DNA methylation profiling by reduced representation bisulfite sequencing (RRBS)

The Bisulfite Analysis Toolkit (BAT) (Kretzmer et al., 2017) was used for RRBS analysis. Prior to analysis, raw data was quality trimmed with TrimGalore! version 0.6.6 (Martin, 2011). Trimming was performed on non-directional (--non_directional) paired-end reads (--paired). An additional 2 bp were trimmed from the 3′ end of the first read and 5′ end of the second read (--rrbs). Sequence quality was assessed with FastQC version 0.11.9 (Andrews, 2010) and MultiQC version 1.11 (Ewels et al., 2016) after trimming.

Trimmed paired reads were aligned to the genome using BAT_mapping module specifying non-directional input (-F 2). Mapping statistics and methylation calling was done using BAT_mapping_stat and BAT_calling modules, respectively. CpG methylation data was filtered to retain only a minimum 10 and maximum 500 reads per sample (--MD_min 10, –MD_max 500 --CG). The data were sorted using bedGraphs (sortBed version 2.29.1; Quinlan and Hall, 2010) and merged into treatment-specific groups (BAT_summarize). Within each group, one sample was allowed to have missing data for a CpG locus (--mis1 1, --mis2 1). If data were missing for more than one sample at a particular CpG, it was not included in the downstream analysis. Chromosome lengths were specified (--cs) for merging methylation information accurately. BAT_overview was used to obtain average methylation rate per sample in each group, hierarchical clustering of sample methylation rates, distribution of CpG methylation, comparison of methylation rate between groups for common loci, and differences in mean methylation rate between groups. Differentially methylated regions (DMR) — defined as regions with at least 10 CpGs, a minimum methylation rate difference of 0.1, and q-value <0.05 were identified for each comparison using BAT_DMRcalling module. The closest genome feature to each DMR was characterized using closestBed.

## Supplementary Material

10.1242/biolopen.061801_sup1 Supplementary information

## References

[BIO061801C1] Able, K. W., Hagan, S. M., Kovitvongsa, K., Brown, S. A. and Lamonaca, J. C. (2007). Piscivory by the mummichog (Fundulus heteroclitus): Evidence from the laboratory and salt marshes. *J. Exp. Mar. Biol. Ecol.* 345, 26-37. 10.1016/j.jembe.2007.01.003

[BIO061801C2] Aluru, N., Karchner, S. I., Krick, K. S., Zhu, W. and Liu, J. (2018). Role of DNA methylation in altered gene expression patterns in adult zebrafish (Danio rerio) exposed to 3, 3', 4, 4', 5-pentachlorobiphenyl (PCB 126). *Environ. Epigenet.* 4, dvy005. 10.1093/eep/dvy005 29686887 PMC5905506

[BIO061801C3] Anders, S., Pyl, P. T. and Huber, W. (2015). HTSeq--a Python framework to work with high-throughput sequencing data. *Bioinformatics* 31, 166-169. 10.1093/bioinformatics/btu638 25260700 PMC4287950

[BIO061801C4] Andrews, S. (2010). FastQC: A Quality Control Tool for High Throughput Sequence Data [Online]. Available online at: http://www.bioinformatics.babraham.ac.uk/projects/fastqc/.

[BIO061801C5] Beck, B. H., Fuller, S. A., Li, C., Green, B. W., Zhao, H., Rawles, S. D., Webster, C. D. and Peatman, E. (2016). Hepatic transcriptomic and metabolic responses of hybrid striped bass (Morone saxatilis×Morone chrysops) to acute and chronic hypoxic insult. *Comp. Biochem. Physiol. D Genom. Proteomics* 18, 1-9. 10.1016/j.cbd.2016.01.005 26851735

[BIO061801C6] Bello, S. M., Franks, D. G., Stegeman, J. J. and Hahn, M. E. (2001). Acquired resistance to Ah receptor agonists in a population of Atlantic killifish (Fundulus heteroclitus) inhabiting a marine superfund site: in vivo and in vitro studies on the inducibility of xenobiotic metabolizing enzymes. *Toxicol. Sci.* 60, 77-91. 10.1093/toxsci/60.1.77 11222875

[BIO061801C7] Ben Dhaou, C., Mandi, K., Frye, M., Acheampong, A., Radi, A., De Becker, B., Antoine, M., Baeyens, N., Wittamer, V. and Parmentier, M. (2022). Chemerin regulates normal angiogenesis and hypoxia-driven neovascularization. *Angiogenesis* 25, 159-179. 10.1007/s10456-021-09818-1 34524600 PMC9054887

[BIO061801C8] Bertout, J. A., Patel, S. A. and Simon, M. C. (2008). The impact of O2 availability on human cancer. *Nat. Rev. Cancer* 8, 967-975. 10.1038/nrc2540 18987634 PMC3140692

[BIO061801C9] Bogan, S. N. and Yi, S. V. (2024). Potential role of DNA methylation as a driver of plastic responses to the environment across cells, organisms, and populations. *Genome Biol. Evol.* 16, evae022. 10.1093/gbe/evae022 38324384 PMC10899001

[BIO061801C10] Bolger, A. M., Lohse, M. and Usadel, B. (2014). Trimmomatic: a flexible trimmer for Illumina sequence data. *Bioinformatics* 30, 2114-2120. 10.1093/bioinformatics/btu170 24695404 PMC4103590

[BIO061801C11] Borowiec, B. G. and Scott, G. R. (2020). Hypoxia acclimation alters reactive oxygen species homeostasis and oxidative status in estuarine killifish (Fundulus heteroclitus). *J. Exp. Biol.* 223, jeb222877. 10.1242/jeb.222877 32457064

[BIO061801C12] Borowiec, B. G. and Scott, G. R. (2021). Rapid and reversible modulation of blood haemoglobin content during diel cycles of hypoxia in killifish (Fundulus heteroclitus). *Comp. Biochem. Physiol. A Mol. Integr. Physiol.* 261, 111054. 10.1016/j.cbpa.2021.111054 34384878

[BIO061801C13] Borowiec, B. G., Darcy, K. L., Gillette, D. M. and Scott, G. R. (2015). Distinct physiological strategies are used to cope with constant hypoxia and intermittent hypoxia in killifish (Fundulus heteroclitus). *J. Exp. Biol.* 218, 1198-1211. 25722002 10.1242/jeb.114579

[BIO061801C14] Borowiec, B. G., McClelland, G. B., Rees, B. B. and Scott, G. R. (2018). Distinct metabolic adjustments arise from acclimation to constant hypoxia and intermittent hypoxia in estuarine killifish (Fundulus heteroclitus). *J. Exp. Biol.* 221, jeb190900. 10.1242/jeb.190900 30518600

[BIO061801C15] Borowiec, B. G., Hoffman, R. D., Hess, C. D., Galvez, F. and Scott, G. R. (2020). Interspecific variation in hypoxia tolerance and hypoxia acclimation responses in killifish from the family Fundulidae. *J. Exp. Biol.* 223, jeb209692. 10.1242/jeb.209692 31988166 PMC7044458

[BIO061801C16] Cai, W., Xiong Chen, Z., Rane, G., Satendra Singh, S., Choo, Z., Wang, C., Yuan, Y., Zea Tan, T., Arfuso, F., Yap, C. T. et al. (2017). Wanted DEAD/H or alive: helicases winding up in cancers. *J. Natl. Cancer Inst.* 109, djw278. 10.1093/jnci/djw278 28122908

[BIO061801C17] Chan, J. and Vandeberg, J. L. (2012). Hepatobiliary transport in health and disease. *Clin. Lipidol.* 7, 189-202. 10.2217/clp.12.12 22859919 PMC3408080

[BIO061801C18] Chan, W. K., Yao, G., Gu, Y. Z. and Bradfield, C. A. (1999). Cross-talk between the aryl hydrocarbon receptor and hypoxia inducible factor signaling pathways. Demonstration of competition and compensation. *J. Biol. Chem.* 274, 12115-12123. 10.1074/jbc.274.17.12115 10207038

[BIO061801C19] Chee, N. T., Lohse, I. and Brothers, S. P. (2019). mRNA-to-protein translation in hypoxia. *Mol. Cancer* 18, 49. 10.1186/s12943-019-0968-4 30925920 PMC6441220

[BIO061801C20] Chen, G., Pang, M., Yu, X., Wang, J. and Tong, J. (2021). Transcriptome sequencing provides insights into the mechanism of hypoxia adaption in bighead carp (Hypophthalmichthys nobilis). *Comp. Biochem. Physiol. D Genomics Proteomics* 40, 100891. 10.1016/j.cbd.2021.100891 34404015

[BIO061801C21] Deaton, A. M. and Bird, A. (2011). CpG islands and the regulation of transcription. *Genes Dev.* 25, 1010-1022. 10.1101/gad.2037511 21576262 PMC3093116

[BIO061801C22] del Peso, L., Castellanos, M. C., Temes, E., Martin-Puig, S., Cuevas, Y., Olmos, G. and Landazuri, M. O. (2003). The von Hippel Lindau/hypoxia-inducible factor (HIF) pathway regulates the transcription of the HIF-proline hydroxylase genes in response to low oxygen. *J. Biol. Chem.* 278, 48690-48695. 10.1074/jbc.M308862200 14506252

[BIO061801C23] Dobin, A. and Gingeras, T. R. (2015). Mapping RNA-seq reads with STAR. *Curr. Protoc. Bioinformatics* 51, 11.14.11-11.14.19. 10.1002/0471250953.bi1114s51 PMC4631051 26334920

[BIO061801C24] Du, S. N., Mahalingam, S., Borowiec, B. G. and Scott, G. R. (2016). Mitochondrial physiology and reactive oxygen species production are altered by hypoxia acclimation in killifish (Fundulus heteroclitus). *J. Exp. Biol.* 219, 1130-1138. 26896545 10.1242/jeb.132860

[BIO061801C25] Ewels, P., Magnusson, M., Lundin, S. and Kaller, M. (2016). MultiQC: summarize analysis results for multiple tools and samples in a single report. *Bioinformatics* 32, 3047-3048. 10.1093/bioinformatics/btw354 27312411 PMC5039924

[BIO061801C26] Feng, C., Li, X., Sha, H., Luo, X., Zou, G. and Liang, H. (2022). Comparative transcriptome analysis provides novel insights into the molecular mechanism of the silver carp (Hypophthalmichthys molitrix) brain in response to hypoxia stress. *Comp. Biochemi. Physiol. Part D Genomics Proteomics* 41, 100951. 10.1016/j.cbd.2021.100951 34923202

[BIO061801C27] Flight, P. A., Nacci, D., Champlin, D., Whitehead, A. and Rand, D. M. (2011). The effects of mitochondrial genotype on hypoxic survival and gene expression in a hybrid population of the killifish, Fundulus heteroclitus. *Mol. Ecol.* 20, 4503-4520. 10.1111/j.1365-294X.2011.05290.x 21980951 PMC3292440

[BIO061801C28] Fong, G. H. and Takeda, K. (2008). Role and regulation of prolyl hydroxylase domain proteins. *Cell Death Differ.* 15, 635-641. 10.1038/cdd.2008.10 18259202

[BIO061801C29] Gilkes, D. M., Semenza, G. L. and Wirtz, D. (2014). Hypoxia and the extracellular matrix: drivers of tumour metastasis. *Nat. Rev. Cancer* 14, 430-439. 10.1038/nrc3726 24827502 PMC4283800

[BIO061801C30] Gu, Y., Jin, C. X., Tong, Z. H., Jiang, T., Yao, F. C., Zhang, Y., Huang, J., Song, F. B., Sun, J. L. and Luo, J. (2024). Expression of genes related to gonadal development and construction of gonadal DNA methylation maps of Trachinotus blochii under hypoxia. *Sci. Total Environ.* 935, 173172. 10.1016/j.scitotenv.2024.173172 38740210

[BIO061801C31] Guan, L., Ge, R. and Ma, S. (2024). Newsights of endoplasmic reticulum in hypoxia. *Biomed. Pharmacother.* 175, 116812. 10.1016/j.biopha.2024.116812 38781866

[BIO061801C32] Harishchandra, A., Di Giulio, R. T. and Jayasundara, N. (2024). Transcriptomic and methylomic analyses show significant shifts in biosynthetic processes and reduced intrapopulation gene expression variance in PAH-Adapted atlantic killifish. *Environ. Sci. Technol.* 58, 20859-20872. 10.1021/acs.est.4c06845 39552013 PMC11651382

[BIO061801C33] Hoffman, M. A., Ohh, M., Yang, H., Klco, J. M., Ivan, M. and Kaelin, W. G., Jr. (2001). von Hippel-Lindau protein mutants linked to type 2C VHL disease preserve the ability to downregulate HIF. *Hum. Mol. Genet.*, 10: 1019-1027. 10.1093/hmg/10.10.1019 11331612

[BIO061801C34] Ivan, M. and Kaelin, W. G. Jr (2017). The EGLN-HIF O(2)-sensing system: multiple inputs and feedbacks. *Mol. Cell* 66, 772-779. 10.1016/j.molcel.2017.06.002 28622522 PMC5613951

[BIO061801C35] Janczy-Cempa, E., Mazuryk, O., Kania, A. and Brindell, M. (2022). Significance of specific oxidoreductases in the design of hypoxia-activated prodrugs and fluorescent turn off-on probes for hypoxia imaging. *Cancers (Basel)* 14, 2686. 10.3390/cancers14112686 35681666 PMC9179281

[BIO061801C36] Jaskiewicz, M., Moszynska, A., Kroliczewski, J., Cabaj, A., Bartoszewska, S., Charzynska, A., Gebert, M., Dabrowski, M., Collawn, J. F. and Bartoszewski, R. (2022a). The transition from HIF-1 to HIF-2 during prolonged hypoxia results from reactivation of PHDs and HIF1A mRNA instability. *Cell. Mol. Biol. Lett.* 27, 109. 10.1186/s11658-022-00408-7 36482296 PMC9730601

[BIO061801C37] Jaskiewicz, M., Moszynska, A., Serocki, M., Kroliczewski, J., Bartoszewska, S., Collawn, J. F. and Bartoszewski, R. (2022b). Hypoxia-inducible factor (HIF)-3a2 serves as an endothelial cell fate executor during chronic hypoxia. *EXCLI J.* 21, 454-469. 35391921 10.17179/excli2021-4622 PMC8983852

[BIO061801C38] Jasperse, L., Di Giulio, R. T. and Jayasundara, N. (2023). Bioenergetic effects of polycyclic aromatic hydrocarbon resistance manifest later in life in offspring of fundulus heteroclitus from the elizabeth river. *Environ. Sci. Technol.* 57, 15806-15815. 10.1021/acs.est.3c03610 37818763 PMC10733968

[BIO061801C39] Jayasundara, N., Fernando, P. W., Osterberg, J. S., Cammen, K. M., Schultz, T. F. and Di Giulio, R. T. (2017). Cost of tolerance: physiological consequences of evolved resistance to inhabit a polluted environment in teleost fish fundulus heteroclitus. *Environ. Sci. Technol.* 51, 8763-8772. 10.1021/acs.est.7b01913 28682633 PMC5745795

[BIO061801C40] Jones, E. R. and Griffitt, R. J. (2022). Oil and hypoxia alter DNA methylation and transcription of genes related to neurological function in larval Cyprinodon variegatus. *Aquat. Toxicol.* 251, 106267. 10.1016/j.aquatox.2022.106267 36058102

[BIO061801C41] Jones, R. B., Dorsett, K. A., Hjelmeland, A. B. and Bellis, S. L. (2018). The ST6Gal-I sialyltransferase protects tumor cells against hypoxia by enhancing HIF-1a signaling. *J. Biol. Chem.* 293, 5659-5667. 10.1074/jbc.RA117.001194 29475939 PMC5900773

[BIO061801C42] Kallio, P. J., Wilson, W. J., O'Brien, S., Makino, Y. and Poellinger, L. (1999). Regulation of the hypoxia-inducible transcription factor 1alpha by the ubiquitin-proteasome pathway. *J. Biol. Chem.* 274, 6519-6525. 10.1074/jbc.274.10.6519 10037745

[BIO061801C43] Karchner, S. I., Powell, W. H. and Hahn, M. E. (1999). Identification and functional characterization of two highly divergent aryl hydrocarbon receptors (AHR1 and AHR2) in the teleost Fundulus heteroclitus. Evidence for a novel subfamily of ligand-binding basic helix loop helix-Per-ARNT-Sim (bHLH-PAS) factors. *J. Biol. Chem.* 274, 33814-33824. 10.1074/jbc.274.47.33814 10559277

[BIO061801C44] Kelly, T., Johnsen, H., Burgerhout, E., Tveiten, H., Thesslund, T., Andersen, O. and Robinson, N. (2020). Low oxygen stress during early development influences regulation of hypoxia-response genes in farmed atlantic salmon (Salmo salar). *G3 (Bethesda)* 10, 3179-3188. 10.1534/g3.120.401459 32636218 PMC7466997

[BIO061801C45] Kimball, M. E. and Able, K. W. (2007). Nekton utilization of intertidal salt marsh creeks: Tidal influences in natural Spartina, invasive Phragmites, and marshes treated for Phragmites removal. *J. Exp. Mar. Biol. Ecol.* 346, 87-101. 10.1016/j.jembe.2007.03.006

[BIO061801C46] Kindrick, J. D. and Mole, D. R. (2020). Hypoxic regulation of gene transcription and chromatin: cause and effect. *Int. J. Mol. Sci.* 21, 8320. 10.3390/ijms21218320 33171917 PMC7664190

[BIO061801C47] Kipp, H. and Arias, I. M. (2002). Trafficking of canalicular ABC transporters in hepatocytes. *Annu. Rev. Physiol.* 64, 595-608. 10.1146/annurev.physiol.64.081501.155793 11826281

[BIO061801C102] Klassen, R. V., Stroeder, J., Coussen, F., Hafner, A. S., Petersen, J. D., Renancio, C., Schmitz, L. J., Normand, E., Lodder, J. C., Rotaru, D. C., Rao-Ruiz, P., Spijker, S., Mansvelder, H. D., Choquet, D. and Smit, A. B. (2016). Shisa6 traps AMPA receptors at postsynaptic sites and prevents their desensitization during synaptic activity. *Nat Commun.* 7, 10682. 26931375 10.1038/ncomms10682 PMC4778035

[BIO061801C48] Kraemer, L. D. and Schulte, P. M. (2004). Prior PCB exposure suppresses hypoxia-induced up-regulation of glycolytic enzymes in Fundulus heteroclitus. *Comp. Biochem. Physiol. C Toxicol. Pharmacol.* 139, 23-29. 10.1016/j.cca.2004.08.015 15556062

[BIO061801C49] Kretzmer, H., Otto, C. and Hoffmann, S. (2017). BAT: Bisulfite Analysis Toolkit: BAT is a toolkit to analyze DNA methylation sequencing data accurately and reproducibly. It covers standard processing and analysis steps from raw read mapping up to annotation data integration and calculation of correlating DMRs. *F1000Res* 6, 1490. 10.12688/f1000research.12302.1 28979767 PMC5590080

[BIO061801C50] Lau, S. C., Mehdi, H., Bragg, L. M., Servos, M. R., Balshine, S. and Scott, G. R. (2021). Exposure to wastewater effluent disrupts hypoxia responses in killifish (Fundulus heteroclitus). *Environ. Pollut.* 284, 117373. 10.1016/j.envpol.2021.117373 34077896

[BIO061801C51] Lee, H. Y., Choi, K., Oh, H., Park, Y. K. and Park, H. (2014). HIF-1-dependent induction of Jumonji domain-containing protein (JMJD) 3 under hypoxic conditions. *Mol. Cells* 37, 43-50. 10.14348/molcells.2014.2250 24552709 PMC3907005

[BIO061801C52] Li, F. and Ding, J. (2019). Sialylation is involved in cell fate decision during development, reprogramming and cancer progression. *Protein Cell* 10, 550-565. 10.1007/s13238-018-0597-5 30478534 PMC6626595

[BIO061801C53] Li, L., Shen, S., Bickler, P., Jacobson, M. P., Wu, L. F. and Altschuler, S. J. (2023). Searching for molecular hypoxia sensors among oxygen-dependent enzymes. *eLife* 12, e87705. 10.7554/eLife.87705 37494095 PMC10371230

[BIO061801C54] Lindberg, C. D., Jayasundara, N., Kozal, J. S., Leuthner, T. C. and Di Giulio, R. T. (2017). Resistance to polycyclic aromatic hydrocarbon toxicity and associated bioenergetic consequences in a population of Fundulus heteroclitus. *Ecotoxicology* 26, 435-448. 10.1007/s10646-017-1775-6 28213827 PMC5398948

[BIO061801C55] Lindner, M., Verhagen, I., Viitaniemi, H. M., Laine, V. N., Visser, M. E., Husby, A. and van Oers, K. (2021). Temporal changes in DNA methylation and RNA expression in a small song bird: within- and between-tissue comparisons. *BMC Genomics* 22, 36. 10.1186/s12864-020-07329-9 33413102 PMC7792223

[BIO061801C56] Lisy, K. and Peet, D. J. (2008). Turn me on: regulating HIF transcriptional activity. *Cell Death Differ.* 15, 642-649. 10.1038/sj.cdd.4402315 18202699

[BIO061801C57] Luo, Z., Tian, M., Yang, G., Tan, Q., Chen, Y., Li, G., Zhang, Q., Li, Y., Wan, P. and Wu, J. (2022). Hypoxia signaling in human health and diseases: implications and prospects for therapeutics. *Signal. Transduct. Target Ther.* 7, 218. 10.1038/s41392-022-01080-1 35798726 PMC9261907

[BIO061801C58] Martin, M. (2011). Cutadapt removes adapter sequences from high-throughput sequencing reads. *EMBnetjournal* 17, 10-12.

[BIO061801C59] Masuda, K., Abdelmohsen, K. and Gorospe, M. (2009). RNA-binding proteins implicated in the hypoxic response. *J. Cell. Mol. Med.* 13, 2759-2769. 10.1111/j.1582-4934.2009.00842.x 19583805 PMC2832090

[BIO061801C60] Mitchell, P. and Tollervey, D. (2001). mRNA turnover. *Curr. Opin. Cell Biol.* 13, 320-325. 10.1016/S0955-0674(00)00214-3 11343902

[BIO061801C61] Moszynska, A., Jaskiewicz, M., Serocki, M., Cabaj, A., Crossman, D. K., Bartoszewska, S., Gebert, M., Dabrowski, M., Collawn, J. F. and Bartoszewski, R. (2022). The hypoxia-induced changes in miRNA-mRNA in RNA-induced silencing complexes and HIF-2 induced miRNAs in human endothelial cells. *FASEB J.* 36, e22412. 10.1096/fj.202101987R 35713587 PMC9220987

[BIO061801C62] Mu, Y., Li, W., Wei, Z., He, L., Zhang, W. and Chen, X. (2020). Transcriptome analysis reveals molecular strategies in gills and heart of large yellow croaker (Larimichthys crocea) under hypoxia stress. *Fish Shellfish Immunol.* 104, 304-313. 10.1016/j.fsi.2020.06.028 32544557

[BIO061801C63] Murphy, T. E., Harris, J. C. and Rees, B. B. (2023). Hypoxia-inducible factor 1 alpha protein increases without changes in mRNA during acute hypoxic exposure of the Gulf killifish, Fundulus grandis. *Biol. Open* 12, bio060167. 10.1242/bio.060167 38116983 PMC10805151

[BIO061801C64] Naba, A., Clauser, K. R., Hoersch, S., Liu, H., Carr, S. A. and Hynes, R. O. (2012). The matrisome: in silico definition and in vivo characterization by proteomics of normal and tumor extracellular matrices. *Mol. Cell. Proteomics* 11, M111.014647. 10.1074/mcp.M111.014647 PMC3322572 22159717

[BIO061801C65] Nacci, D. E., Champlin, D. and Jayaraman, S. (2010). Adaptation of the Estuarine Fish Fundulus heteroclitus (Atlantic Killifish) to Polychlorinated Biphenyls (PCBs). *Estuaries Coasts* 33, 853-864. 10.1007/s12237-009-9257-6

[BIO061801C66] Nie, M., Blankenship, A. L. and Giesy, J. P. (2001). Interactions between aryl hydrocarbon receptor (AhR) and hypoxia signaling pathways. *Environ. Toxicol. Pharmacol.* 10, 17-27. 10.1016/S1382-6689(01)00065-5 11382553

[BIO061801C67] Oksanen, J., Simpson, G., Blanchet, F., Kindt, R., Legendre, P., Minchin, P., O'hara, R., Solymos, P., Stevens, M., Szoecs, E., Wagner, H., Barbour, M., Bedward, M., Bolker, B., Borcard, D., Carvalho, G., Chirico, M., De Caceres, M., Durand, S., Evangelista, H., FitzJohn, R., Friendly, M., Furneaux, B., Hannigan, G., Hill, M., Lahti, L., McGlinn, D., Ouellette, M., Ribeiro Cunha, E., Smith, T., Stier, A., Ter Braak, C. and Weedon, J. vegan: Community Ecology Package. R package version 2.6–5 ed. https://github.com/vegandevs/vegan2023.

[BIO061801C68] Patak, P., Jin, F., Schafer, S. T., Metzen, E. and Hermann, D. M. (2011). The ATP-binding cassette transporters ABCB1 and ABCC1 are not regulated by hypoxia in immortalised human brain microvascular endothelial cells. *Exp. Transl. Stroke Med.* 3, 12. 10.1186/2040-7378-3-12 22029974 PMC3213079

[BIO061801C69] Qin, H., Zhang, X., Xie, T., Gao, Y., Li, J. and Jia, Y. (2023). Hepatic transcriptomic analysis reveals that Hif1α/ldha signal is involved in the regulation of hypoxia stress in black rockfish Sebastes schlegelii. *Comp. Biochem. Physiol. Part D: Genomics and Proteomics\* 47, 101098. 10.1016/j.cbd.2023.101098 37229966

[BIO061801C70] Quinlan, A. R. and Hall, I. M. (2010). BEDTools: a flexible suite of utilities for comparing genomic features. *Bioinformatics* 26, 841-842. 10.1093/bioinformatics/btq033 20110278 PMC2832824

[BIO061801C71] Ragsdale, A., Ortega-Recalde, O., Dutoit, L., Besson, A. A., Chia, J. H. Z., King, T., Nakagawa, S., Hickey, A., Gemmell, N. J., Hore, T. et al. (2022). Paternal hypoxia exposure primes offspring for increased hypoxia resistance. *BMC Biol.* 20, 185. 10.1186/s12915-022-01389-x 36038899 PMC9426223

[BIO061801C72] Reid, N. M., Proestou, D. A., Clark, B. W., Warren, W. C., Colbourne, J. K., Shaw, J. R., Karchner, S. I., Hahn, M. E., Nacci, D., Oleksiak, M. F. et al. (2016). The genomic landscape of rapid repeated evolutionary adaptation to toxic pollution in wild fish. *Science* 354, 1305-1308. 10.1126/science.aah4993 27940876 PMC5206662

[BIO061801C73] Ridgway, M. R. and Scott, G. R. (2023). Constant temperature and fluctuating temperature have distinct effects on hypoxia tolerance in killifish (Fundulus heteroclitus). *J. Exp. Biol.* 226, jeb245425. 10.1242/jeb.245425 37073679

[BIO061801C74] Robinson, C. M., Neary, R., Levendale, A., Watson, C. J. and Baugh, J. A. (2012). Hypoxia-induced DNA hypermethylation in human pulmonary fibroblasts is associated with Thy-1 promoter methylation and the development of a pro-fibrotic phenotype. *Respir. Res.* 13, 74. 10.1186/1465-9921-13-74 22938014 PMC3519562

[BIO061801C75] Robinson, M. D., McCarthy, D. J. and Smyth, G. K. (2010). edgeR: a Bioconductor package for differential expression analysis of digital gene expression data. *Bioinformatics* 26, 139-140. 10.1093/bioinformatics/btp616 19910308 PMC2796818

[BIO061801C76] Rojas, M., Salvatierra, R., Smok, C., Sandoval, C., Souza-Mello, V. and del Sol, M. (2024). Effect of hypoxia on the post-hatching growth of the body of the fry and the caudal fin of the Atlantic Salmon (Salmo salar). *Front. Mar. Sci.* 11, 1425671. 10.3389/fmars.2024.1425671

[BIO061801C77] Rosa, Ng, M. and Jain, R. K. (2016). Hypoxia-induced DNA hypermethylation: another reason to normalize tumor vessels. *Transl. Cancer Res.* 5, S1358-S1362. 10.21037/tcr.2016.12.72

[BIO061801C78] Salminen, A., Kaarniranta, K. and Kauppinen, A. (2016). Hypoxia-inducible histone lysine demethylases: impact on the aging process and age-related diseases. *Aging Dis.* 7, 180-200. 10.14336/AD.2015.0929 27114850 PMC4809609

[BIO061801C79] Sayols, S. (2023). rrvgo: a Bioconductor package for interpreting lists of Gene Ontology terms. *MicroPubl. Biol.* 10.17912/micropub.biology.000811 PMC10155054 37151216

[BIO061801C80] Seta, K. A., Yuan, Y., Spicer, Z., Lu, G., Bedard, J., Ferguson, T. K., Pathrose, P., Cole-Strauss, A., Kaufhold, A. and Millhorn, D. E. (2004). The role of calcium in hypoxia-induced signal transduction and gene expression. *Cell Calcium* 36, 331-340. 10.1016/j.ceca.2004.02.006 15261489

[BIO061801C81] Shang, F., Bao, M., Liu, F., Hu, Z., Wang, S., Yang, X., Yu, Y., Zhang, H., Jiang, C., Qiu, X. et al. (2022a). Transcriptome profiling of tiger pufferfish (Takifugu rubripes) gills in response to acute hypoxia. *Aquaculture* 557, 738324. 10.1016/j.aquaculture.2022.738324

[BIO061801C82] Shang, F., Lu, Y., Li, Y., Han, B., Wei, R., Liu, S., Liu, Y., Liu, Y. and Wang, X. (2022b). Transcriptome analysis identifies key metabolic changes in the brain of Takifugu rubripes in response to chronic hypoxia. *Genes (Basel)* 13, 1347. 10.3390/genes13081347 36011255 PMC9407616

[BIO061801C83] Soliman, S. H. A., Iwanaszko, M., Zheng, B., Gold, S., Howard, B. C., Das, M., Chakrabarty, R. P., Chandel, N. S. and Shilatifard, A. (2024). Transcriptional elongation control of hypoxic response. *Proc. Natl. Acad. Sci. USA* 121, e2321502121. 10.1073/pnas.2321502121 38564636 PMC11009653

[BIO061801C84] Thews, O., Gassner, B., Kelleher, D. K. and Gekle, M. (2008). Activity of drug efflux transporters in tumor cells under hypoxic conditions. *Adv. Exp. Med. Biol.* 614, 157-164. 10.1007/978-0-387-74911-2_19 18290326

[BIO061801C85] Thienpont, B., Steinbacher, J., Zhao, H., D'Anna, F., Kuchnio, A., Ploumakis, A., Ghesquiere, B., Van Dyck, L., Boeckx, B., Schoonjans, L. et al. (2016). Tumour hypoxia causes DNA hypermethylation by reducing TET activity. *Nature* 537, 63-68. 10.1038/nature19081 27533040 PMC5133388

[BIO061801C86] Thomas, P. A. and Kinsey, S. T. (2024). Hypoxia tolerance of two killifish species. *Integr. Comp. Biol.* 64, 1115-1130. 10.1093/icb/icae144 39238158 PMC11518574

[BIO061801C87] Thoral, E., Farhat, E., Roussel, D., Cheng, H., Guillard, L., Pamenter, M. E., Weber, J. M. and Teulier, L. (2022). Different patterns of chronic hypoxia lead to hierarchical adaptive mechanisms in goldfish metabolism. *J. Exp. Biol.* 225, jeb243194. 10.1242/jeb.243194 34881781

[BIO061801C88] Townley, I. K., Karchner, S. I., Skripnikova, E., Wiese, T. E., Hahn, M. E. and Rees, B. B. (2017). Sequence and functional characterization of hypoxia-inducible factors, HIF1alpha, HIF2alphaa, and HIF3alpha, from the estuarine fish, Fundulus heteroclitus. *Am. J. Physiol. Regul. Integr. Comp. Physiol.* 312, R412-R425. 10.1152/ajpregu.00402.2016 28039194 PMC5402000

[BIO061801C89] Vadlapatla, R. K., Vadlapudi, A. D., Ponnaluri, V. K., Pal, D., Mukherji, M. and Mitra, A. K. (2013). Molecular expression and functional activity of efflux and influx transporters in hypoxia induced retinal pigment epithelial cells. *Int. J. Pharm.* 454, 444-452. 10.1016/j.ijpharm.2013.06.044 23827654 PMC3761793

[BIO061801C90] Vorrink, S. U. and Domann, F. E. (2014). Regulatory crosstalk and interference between the xenobiotic and hypoxia sensing pathways at the AhR-ARNT-HIF1alpha signaling node. *Chem. Biol. Interact.* 218, 82-88. 10.1016/j.cbi.2014.05.001 24824450 PMC4091760

[BIO061801C91] Vujić, T., Schvartz, D., Furlani, I. L., Meister, I., Gonzalez-Ruiz, V., Rudaz, S. and Sanchez, J. C. (2022). Oxidative stress and extracellular matrix remodeling are signature pathways of extracellular vesicles released upon morphine exposure on human brain microvascular endothelial cells. *Cells* 11, 3926. 10.3390/cells11233926 36497184 PMC9741159

[BIO061801C92] Wallace, R. B. and Gobler, C. J. (2021). The role of algal blooms and community respiration in controlling the temporal and spatial dynamics of hypoxia and acidification in eutrophic estuaries. *Mar. Pollut. Bull.* 172, 112908. 10.1016/j.marpolbul.2021.112908 34526266

[BIO061801C93] Wang, Y., Li, G., Deng, M., Liu, X., Huang, W., Zhang, Y., Liu, M. and Chen, Y. (2021). The multifaceted functions of RNA helicases in the adaptive cellular response to hypoxia: From mechanisms to therapeutics. *Pharmacol. Ther.* 221, 107783. 10.1016/j.pharmthera.2020.107783 33307143

[BIO061801C94] Wenger, R. H., Kurtcuoglu, V., Scholz, C. C., Marti, H. H. and Hoogewijs, D. (2015). Frequently asked questions in hypoxia research. *Hypoxia (Auckl)* 3, 35-43. 10.2147/HP.S92198 27774480 PMC5045069

[BIO061801C95] Wlcek, K. and Stieger, B. (2014). ATP-binding cassette transporters in liver. *Biofactors* 40, 188-198. 10.1002/biof.1136 24105869

[BIO061801C96] Wouters, B. G. and Koritzinsky, M. (2008). Hypoxia signalling through mTOR and the unfolded protein response in cancer. *Nat. Rev. Cancer* 8, 851-864. 10.1038/nrc2501 18846101

[BIO061801C97] Wu, D. and Yotnda, P. (2011). Induction and testing of hypoxia in cell culture. *J. Vis. Exp.*, e2899. 10.3791/2899 PMC3217626 21860378

[BIO061801C98] Wu, T., Hu, E., Xu, S., Chen, M., Guo, P., Dai, Z., Feng, T., Zhou, L., Tang, W., Zhan, L. et al. (2021). clusterProfiler 4.0: A universal enrichment tool for interpreting omics data. *The Innovation* 2, 100141. 10.1016/j.xinn.2021.100141 34557778 PMC8454663

[BIO061801C99] Yfantis, A., Mylonis, I., Chachami, G., Nikolaidis, M., Amoutzias, G. D., Paraskeva, E. and Simos, G. (2023). Transcriptional response to hypoxia: the role of HIF-1-associated co-regulators. *Cells* 12, 798. 10.3390/cells12050798 36899934 PMC10001186

[BIO061801C100] Zhang, M., Hu, Y., Yang, F., Zhang, J., Zhang, J., Yu, W., Wang, M., Lv, X., Li, J., Bai, T. et al. (2022). Interaction between AhR and HIF-1 signaling pathways mediated by ARNT/HIF-1β. *BMC Pharmacol. Toxicol.* 23, 26. 10.1186/s40360-022-00564-8 35473600 PMC9044668

[BIO061801C101] Zhao, S.-S., Su, X.-L., Pan, R.-J., Lu, L.-Q., Zheng, G.-D. and Zou, S.-M. (2022). The transcriptomic responses of blunt snout bream (Megalobrama amblycephala) to acute hypoxia stress alone, and in combination with bortezomib. *BMC Genomics* 23, 162. 10.1186/s12864-022-08399-7 35216548 PMC8876555

